# Identification of an Immune Classification and Prognostic Genes for Lung Adenocarcinoma Based on Immune Cell Signatures

**DOI:** 10.3389/fmed.2022.855387

**Published:** 2022-03-30

**Authors:** Lili Deng, Fei Long, Ting Wang, Ling Dai, Huajian Chen, Yujun Yang, Guoming Xie

**Affiliations:** ^1^Key Laboratory of Clinical Laboratory Diagnostics (Chinese Ministry of Education), College of Laboratory Medicine, Chongqing Medical University, Chongqing, China; ^2^Chongqing Health Statistics Information Center, Chongqing, China; ^3^Chongqing Emergency Medical Center, Chongqing University Central Hospital, School of Medicine, Chongqing University, Chongqing, China

**Keywords:** lung adenocarcinoma, immune cell signatures, immune subtypes, molecular characteristics, weighted gene correlation network analysis (WGCNA), prognosis

## Abstract

**Objective:**

Current advances in immunotherapy requires accurate tumor sub-classification due to the heterogeneity of lung adenocarcinoma (LUAD). This study aimed to develop a LUAD sub-classification system based on immune cell signatures and identified prognostic gene markers.

**Methods:**

Signatures related to the prognosis of TCGA-LUAD and 4 GSE cohorts were screened and intersected from 184 previously published immune cell signatures. The LUAD samples in the TCGA were clustered by ConsensusClusterPlus. Molecular characteristics, immune characteristics and sensitivity to immunotherapies/chemotherapies were compared. LDA score was established through Linear Discriminant Analysis (LDA). Co-expression module was constructed by Weighted Gene Co-Expression Network Analysis (WGCNA).

**Results:**

Four LUAD subtypes with different molecular and immune characteristics were identified. Significant differences in prognosis among the four subtypes were observed. The IS1 subtype with the worst prognosis showed the highest number of TMB, mutant genes, IFN γ score, angiogenesis score and immune score. Twenty co-expression modules were generated by WGCNA. Blue module, sky blue module and light yellow module were significantly correlated with LUAD prognosis. The hub genes (CCDC90B, ARNTL2, RIPK2, SMCO2 and ADA and NBN) showing great prognostic significance were identified from the blue module. A total of 8 hub genes (NLRC3, CLEC2D, GIMAP5, CXorf65, PARP15, AKNA, ZC3H12D, and ARRDC5) were found in the light yellow module. Except for CXorf65, the expression of the other seven genes were significantly correlated with LUAD prognosis.

**Conclusion:**

This study determined four LUAD subtypes with different molecular and immune characteristics and 13 genes closely related to the prognosis of LUAD. The current findings could help understand the heterogeneity of LUAD immune classes.

## Introduction

Lung cancer was estimated to account for about 1/4 of all cancer deaths in 2021 (1). As the most common type of lung histology, lung adenocarcinoma (LUAD) is characterized by a high heterogeneity at behavioral, cellular and molecular levels, with an overall survival time shorter than 5 years (2). Late diagnosis, limited treatment, recurrence and development of drug resistance are the main challenges for a successful treatment of LUAD (3). Early diagnosis, introduction of new treatments, and overcoming drug resistance are effective in reducing LUAD mortality.

Immunotherapy has greatly changed the direction of LUAD treatment (4). Immunotherapy encourages the host immune system to recognize cancer as a foreign body, stimulating immune system to inhibit cancer cell growth and spread (5). The study of LUAD immunotherapy has many advantages, such as evaluation of pathological responses and anti-tumor immune responses in combination with translational science analysis (6). Immunotherapies include immune modulators, for example, currently interleukin-2 and muramyl tripeptide, dendritic cells, immune checkpoint inhibitors, and engineered T cells have already been used in cancer treatment (7). However, immunotherapy benefits only a small number of patients. The current progress in immunotherapy requires a more accurate sub classification of tumor morphology. LUAD consists of a group of heterogeneous tumors, which can pose a diagnostic challenge, especially when using a small number of biopsy specimens. Clinically, most LUAD can be subclassified using hematoxylin & eosin (H&E) staining to assess histological characteristics. However, in some small biopsy specimens, in addition to morphological evaluation and immunohistochemical features of tumors, the subclassification of tumors is still difficult (8). Growing evidence showed that the subtype classification of LUAD based on gene expression array can provide much information for the molecular characterization and prognosis prediction of LUAD (9). Over the past 20 years, an increasing number of immune cell signatures have been identified, providing a more comprehensive knowledge for various aspects of cancer immunology (10, 11). However, so far, we still lack the study of tumor subtype classification and molecular characterization based on immune cell signatures.

At present, there are many systems biology methods to identify biomarkers related to the prognosis of LUAD and construct gene features. Zhang et al. (12) identified a 7-gene signature in the whole genome using multiomics data set. Guo et al. (13) used genomic instability to identify key lncRNAs for predicting clinical outcomes in patients with lung adenocarcinoma. Lane et al. (14) identified 28 gene markers in the hypoxia related gene expression profile to predict the clinical outcome of non-small cell lung cancer. All the three groups of authors tested their signatures in internal data sets, but they were not used clinically, which means that identifying robust molecular signatures remains a challenge.

In this study, we clustered LUAD samples based on immune cell signatures and identified four different immune subtypes (ISs). We assessed the prognostic differences, transcriptome characteristics, somatic mutation characteristics, immune characteristics, tumor microenvironment characteristics, immunotherapy and drug sensitivity of different among the four ISs, and compared them with the previously established classification. Furthermore, a scoring system was constructed based on Linear Discriminant Analysis (LDA), the modules related to LDA score were screened by WGCNA, and the modules related to the prognosis of LUAD were identified by univariate Cox analysis. Finally, LUAD prognosis-related genes were determined. The ISs we obtained contribute to better understand the heterogeneity of LUAD and the complexity of the immune microenvironment, and highlight the reference value of IS classification for clinical prognosis and treatment decision making. Also, this study identified genes associated with LUAD prognosis that may predict individualized prognosis.

## Materials and Methods

### LUAD Samples Datasets

RNA-Seq data and clinicopathological characteristics of 504 samples of LUAD patients were collected from the TCGA database (https://portal.gdc.cancer.gov/). Microarray profiling dataset GSE37745 (15), GSE19188 (16), GSE50081 (17), GSE30219 (18), and GSE31210 (19) were downloaded from Gene Expression Omnibus (GEO) database (https://www.ncbi.nlm.nih.gov/gds/), and all the five GSE datasets were combined with batch effects removed using the removeBatchEffect of the Limma package (20). After the removal of batch effect, there was no difference in the samples among the GSE datasets through Principal Component Analysis (PCA) (Supplementary Figure 1). The clinical statistics of the samples from TCGA and GEO can be found in Table 1. In addition, we also obtained the exon data set of each sample from TCGA, and calculated the TMB of each patient using R software package maftools (21). Supplementary Figure 2 shows all the workflow of this study.

**Table 1:** Sample clinical statistics for LUAD patients from TCGA and GEO database.

**Clinical Features**		**TCGA-LUAD**	**GEO**
OS	0	321	352
	1	183	230
T Stage	T1	168	
	T2	269	
	T3	45	
	T4	19	
	TX	3	
N Stage	N0	325	
	N1	94	
	N2	71	
	N3	2	
	NX	12	
M Stage	M0	335	
	M1	25	
	MX	144	
Stage	I	270	
	II	119	
	III	81	
	IV	26	
	X	8	
Gender	Male	234	
	Female	270	
Age	≤ 65	247	
	>65	257	

### Immune Cell Signatures

According to Wang et al. (10), we selected previously published 184 cancer-related immune cell signatures to calculate the enrichment scores of samples from different datasets. Survival analysis was performed to screen and intersect the immune cell signatures related to LUAD prognosis in each cohort.

### Consensus Clustering of LUAD Samples

ConsensusClusterPlus (22) was used to cluster 504 LUAD samples in the TCGA cohort. According to the cumulative distribution function (CDF), the optimal number of clusters was determined. The overall survival (OS) of different subtypes was analyzed by Kaplan-Meier Plotter (KM-plotter).

### Molecular Characteristics and Tumor Immune Analyses Between Subgroups

To identify the molecular characteristics of different subtypes, the mutation datasets processed by mutect2 software in TCGA were acquired to analyze the differences in tumor mutation load (TMB) and the number of mutated genes among subgroups. Then the differences in immune checkpoint gene expression among different subgroups were compared. IFN γ scores among subgroups were recorded using Th1/IFN γ gene signatures (23). Mean expression of GZMA and PRF1 (24) were used to assess the intratumoral T cell lytic activity among subgroups. Angiogenesis-associated gene sets (25) were applied to evaluate angiogenesis scores in each subgroup. The scores and degree of immune infiltration of 22 types of immune cells in different subtypes of patients were assessed by CIBERSORT (26). TIDE (http://tide.dfci.harvard.edu/) (27) software also predicted the response of different subgroups to immunotherapy and chemotherapy.

### Construction of Linear Discriminant Analysis (LDA) Model

To better understand the molecular characteristics of LUAD patients, we performed LDA using immune cell signatures with intersected genes to establish a model for evaluating the scores of different subtypes. ROC analysis was performed to determine the specificity and sensitivity of the model.

### Co-expression Module Detection

Weighted Gene Co-Expression Network Analysis (WGCNA) is a biological algorithm for constructing scale-free networks based on gene expression profiles (28). Here, transcripts with discrete value of 50% expression or higher were retained. The soft threshold power was selected by the soft Connectivity function. Based on the expression matrix of LUAD, the adjacency matrix was calculated and converted to topological overlap matrix. Average-linkage hierarchical clustering method was used to cluster genes, and the modules were shown together by a tree with color assignment.

### Pathway Enrichment Analysis for the Modules

Gene Ontology (GO) and Kyoto Encyclopedia of Genes and Genomes (KEGG) analyses were performed using the ClusterProfiler package (29). When there were more than 10 GO terms and pathway enrichments, only the top 10 terms with a *p* < 0.05 were shown.

## Results

### Identification of Four Immune Subgroups Based on Immune Cell Signatures

**Figure 1:**
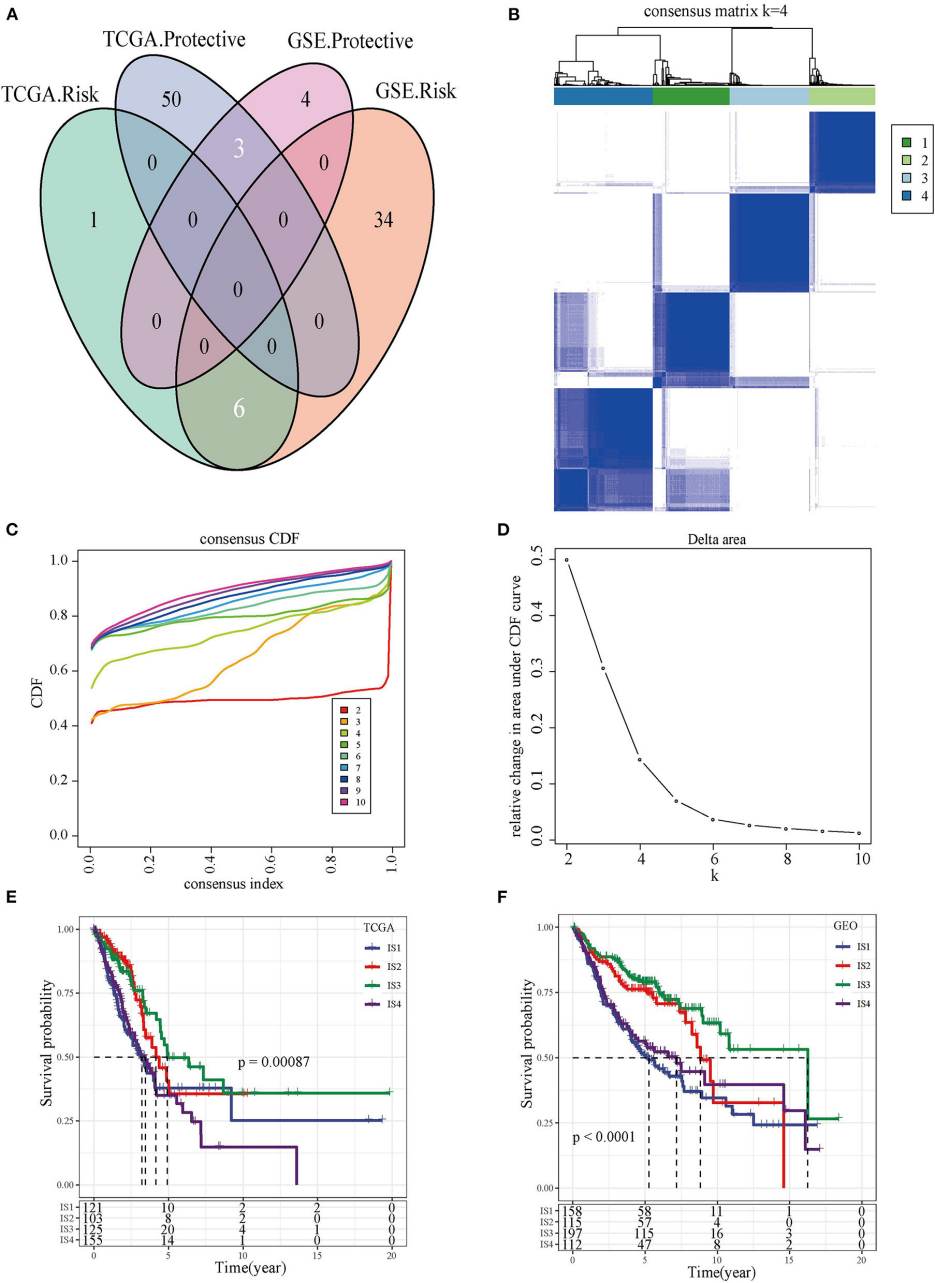
The immune subtypes and survival analysis. **(A)** The Venn diagram showed the intersection of immune cell signatures related to LUAD prognosis in TCGA and GEO databases. **(B)** Heat map of the consensus matrix when the total samples are clustered into four ISs. **(C)** Relationship between the relative changes in the area under the CDF curve and consensus index. **(D)** Heat map of the consensus matrix when the LUAD was clustered into four immune subgroups. **(E)** Kaplan-Meier survival curve showed the OS of four types of ISs patients in TCGA. **(F)** Kaplan-Meier survival curves of four types of ISs patients in GEO.

We found that 60 out of 184 immune cell signatures were significantly correlated with the OS of LUAD by performing univariate Cox analysis. The overlaps in the Venn diagram were immune cell signatures existing in both TCGA and GEO databases and were correlated with LUAD prognosis (Figure 1A). The 504 LUAD samples of TCGA were clustered according to the overlapping prognostic immune cell signatures of the two databases, and the CDF delta area curve showed that CDF plot was relatively stable when the consensus index was 4 (Figures 1B,C). For this reason, LUAD was divided into four immune subgroups (Figure 1D, Supplementary Table 1). Significant differences in prognosis were detected among the four subgroups of ISs whether in TCGA or GEO database (Figures 1E,F, Supplementary Figures 3A,B).

### Molecular Characteristics Analysis Between Four ISs

**Figure 2:**
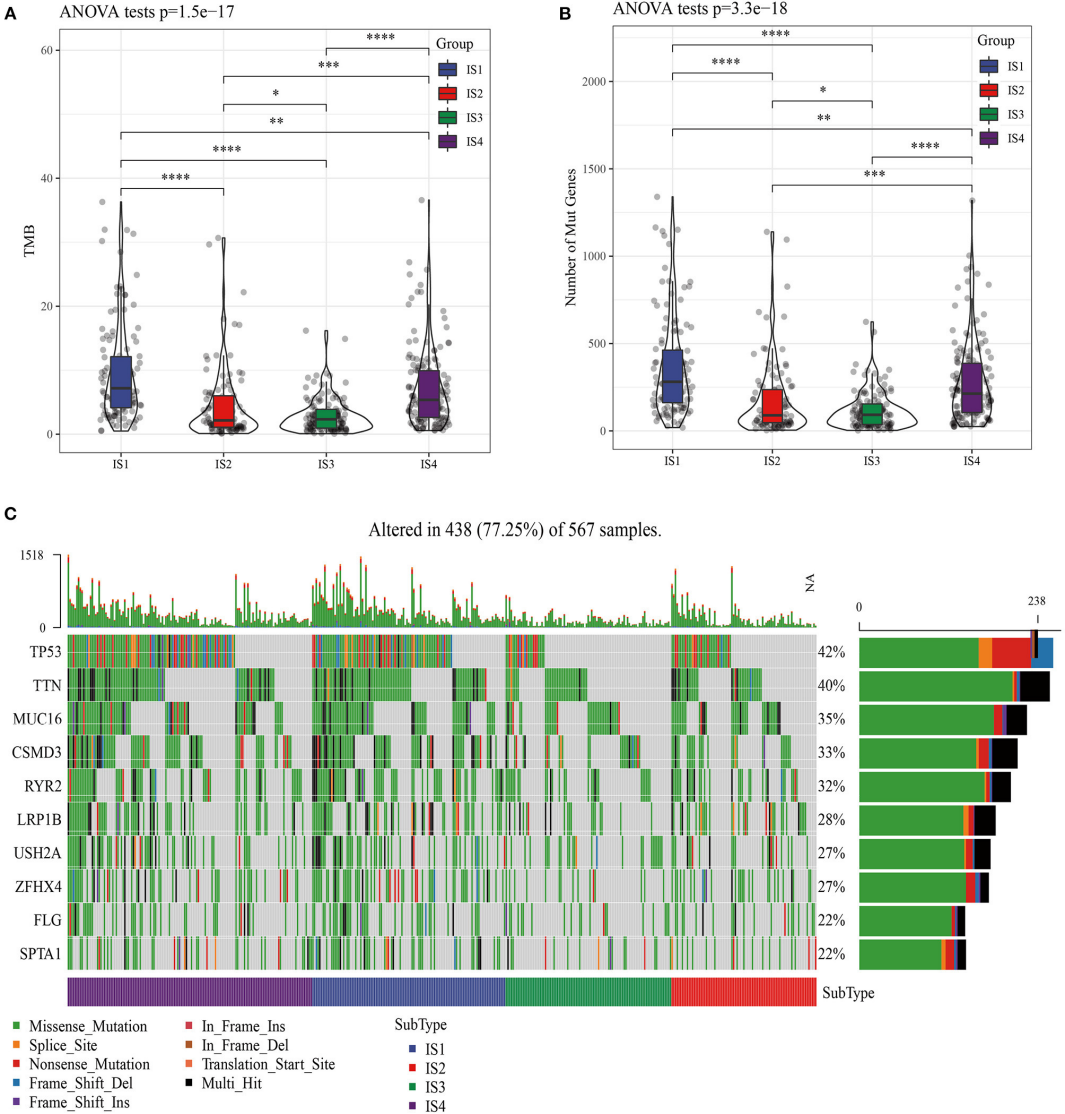
Molecular mutation analysis of four ISs patients in LUAD. **(A)** Four kinds of ISs of LUAD have different TMB. **(B)** Comparison of the number of mutant genes in four kinds of ISs. **(C)** Significant somatic gene mutations in LUAD.

**Figure 3:**
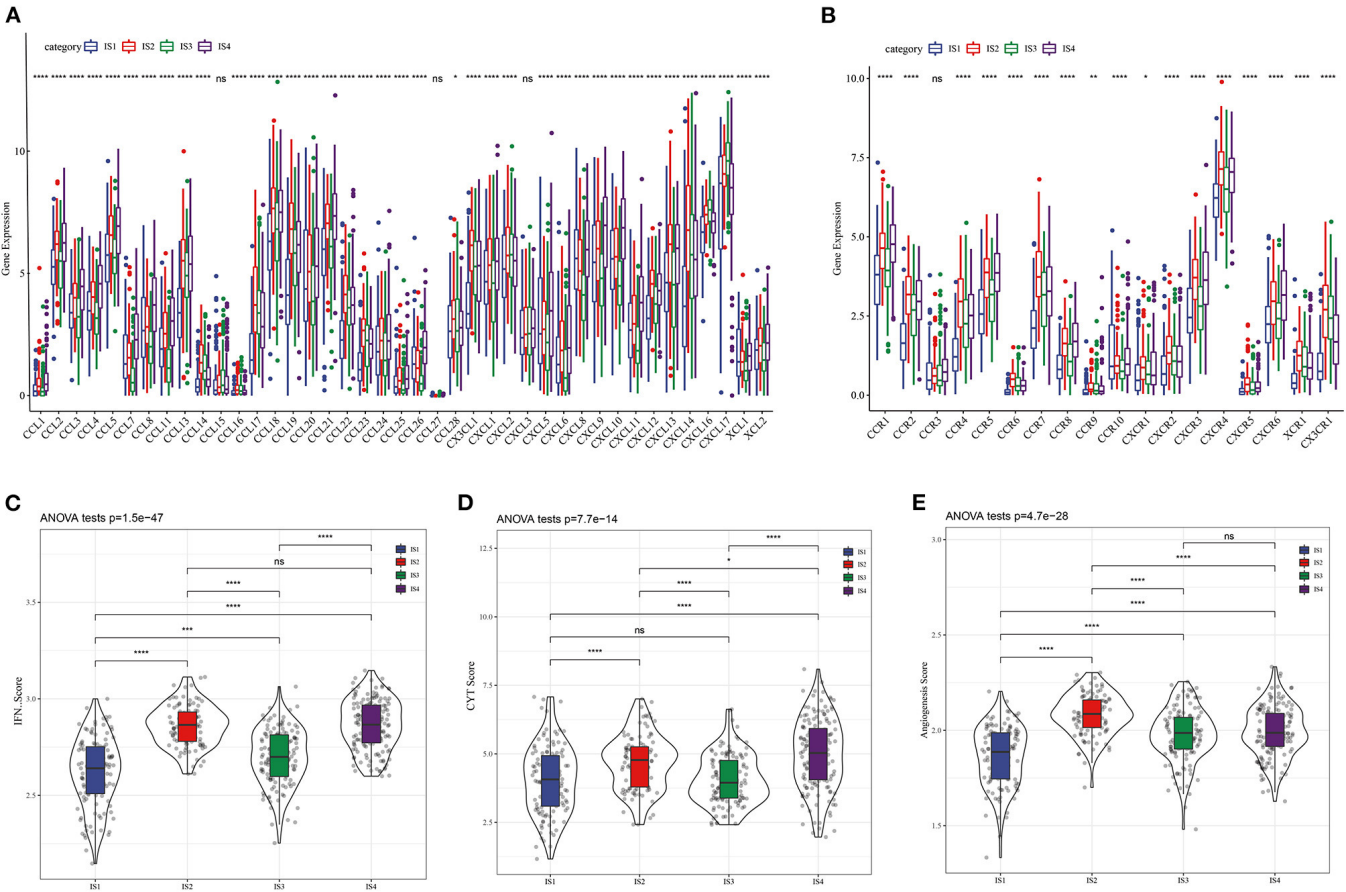
**Four** LUAD subtypes showing different phenotype. **(A)** Expression of chemokine in four LUAD subtypes. **(B)** Expression of chemokine receptors in LUAD subtypes. The difference of **(C)** IFN γ score, **(D)** CYT score, and **(E)** angiogenesis scores among four ISs.

The molecular mutations among the ISs were analyzed to reveal the differences in molecular characteristics of the four ISs, which showed different TMBs and mutant gene numbers. Specifically, IS1 had the highest number of TMB and mutant genes, while IS3 patients with the best prognosis had the lowest number of TMB and mutant genes (Figures 2A,B). Chi-square test identified 10 genes with high frequency of mutation in all ISs, and TP53 mutations were the most common (Figure 2C). Furthermore, the expression of chemokines and chemokine receptors were analyzed in four ISs. More than 90% of the 41 chemokines showed differential expression in the four ISs, and the levels of most chemokines were the lowest in IS1 samples (Figure 3A). The same was also shown in the expression of chemokine receptors (Figure 3B). After examining the differences of IFN γ, CYT and angiogenesis scores in different ISs patients, we found that there were significant differences in IFN γ, CYT and angiogenesis scores in the four types of ISs patients. IS1 showed the lowest IFN γ score and angiogenesis scores, IS4 had the highest CYT score, and IS2 demonstrated the highest angiogenesis scores among the four ISs (Figures 3C–E).

### Cellular Characteristics of Four ISs

**Figure 4:**
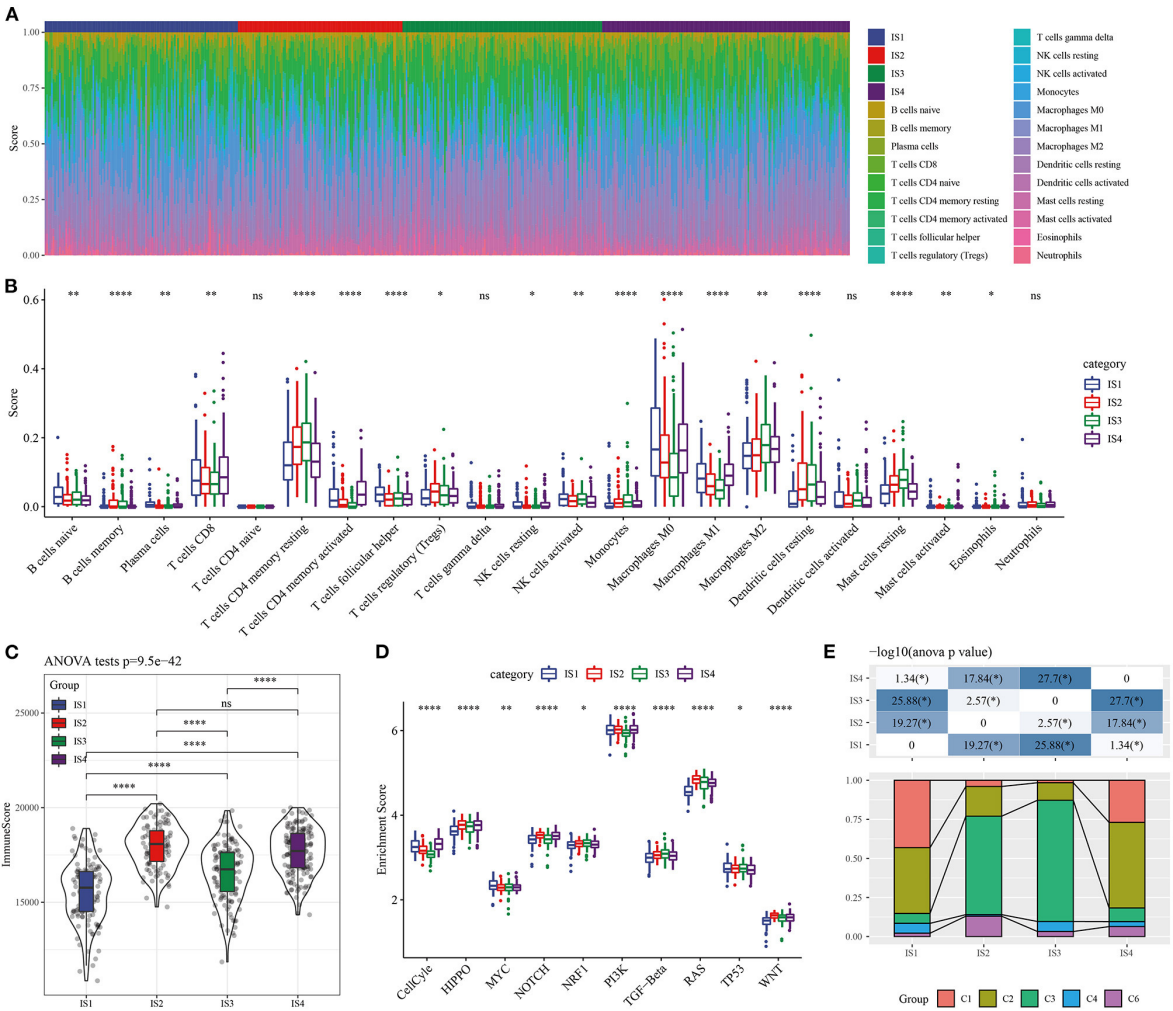
Cellular characteristics of the ISs. **(A)** The distribution of 22 kinds of immune cells in ISs tissue. **(B)** The score of immune cell types with significant differences in two subgroups. **(C)** Distribution of immune scores in the four ISs. **(D)** Enrichment scores of four LUAD subgroups in 10 cancer signaling pathways. **(E)** Distribution of available immunoinfiltrating subtypes molecular subtypes in our subtypes.

As immune system functions critically in tumors, we also explored the relationship between ISs and immune microenvironment. Among the 22 immune cell types examined by the ESTIMATE, except naive CD4 T cells, gamma delta T cells, activated dendritic cells and neutrophils, 18 immune cell displayed notably different scores in IS1-IS4 (Figures 4A,B). Four kinds of ISs also showed different immune scores (Figure 4C). It should be noted that the molecular characteristics of LUAD could be affected by the activation of specific pathways, here four types of ISs patients had significantly different enrichment scores in the 10 typical pathways (cell cycle, Hippo, Myc, Notch, Nrf2, PI3-Kinase/Akt, RTK-RAS, TGF β signaling, p53 and β-catenin/Wnt) (Figure 4D). Distribution of available immune infiltrating subtypes molecular subtypes (30) in our molecular subtypes was analyzed, we found that C1, C2, C3, C4, and C6 subtypes all existed in TCGA data sets but were in different proportion in the four subgroups. C3 subtype has the highest proportion in IS2 and IS3, and the prognosis of these two kinds of ISs patients was better, which, to a certain extent, also confirmed the rationality of the classification of this study (Figure 4E).

### Sensitivity of Different ISs to Immunotherapies/Chemotherapies

**Figure 5:**
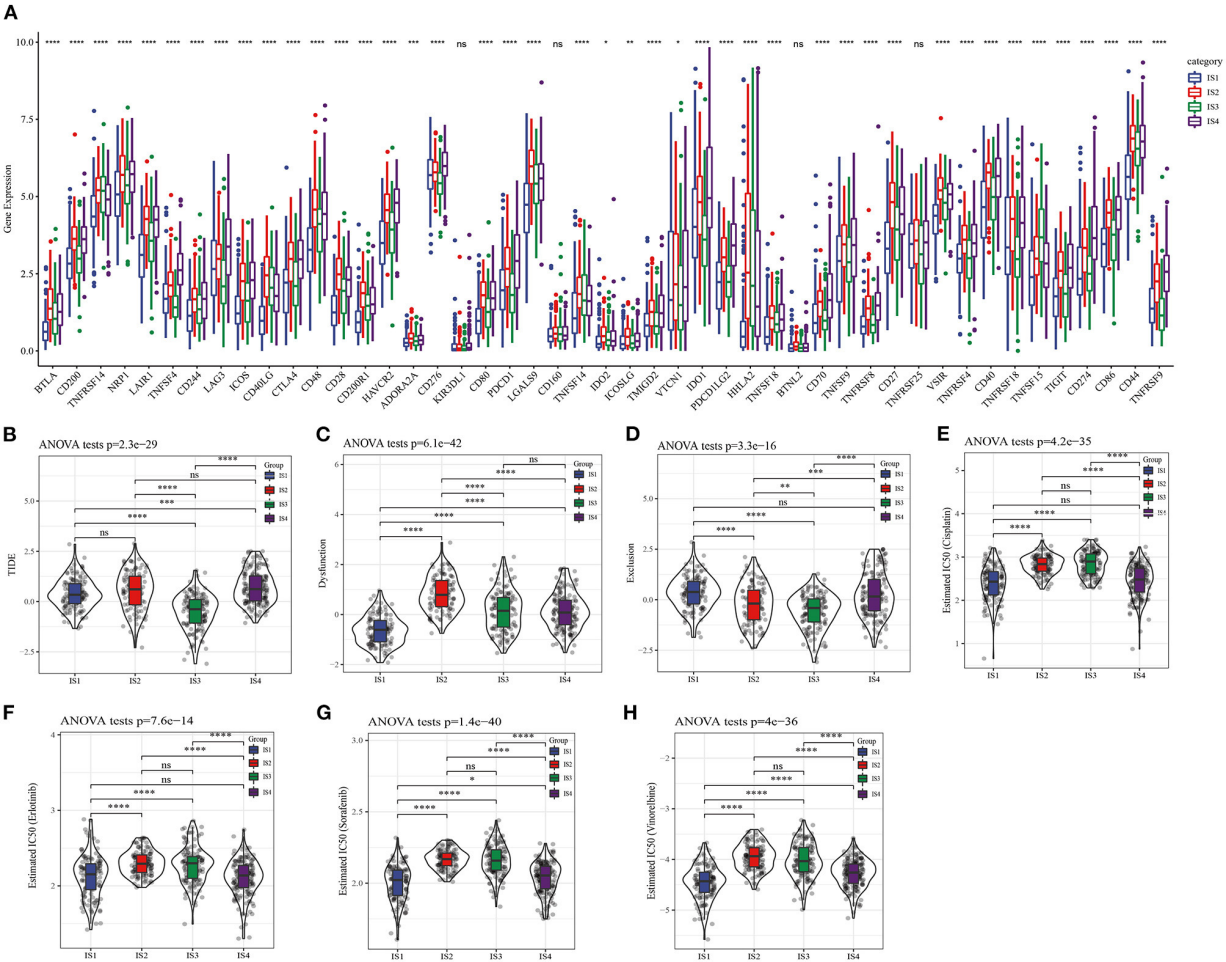
Differential chemotherapeutic and immunotherapeutic responses of ISs. **(A)** Expression of 47 immune checkpoints in different LUAD subtypes. **(B)** The TIDE score of different subtypes. **(C)** T cell dysfunction score of four ISs. **(D)** T cell exclusion score in four subgroups. **(E–H)** All the four ISs had significant differences in the estimated cisplatin, erlotinib, sorafenib and vinorelbine IC_50_ values.

To explore the response of different LUAD subtypes to immunotherapy, the relationship of this immune-related classification of LUAD and immune checkpoint therapy was first analyzed, vast majority of immune checkpoint related genes showed different expression patterns in the four ISs (Figure 5A). As the expression of immune checkpoint is positively correlated with the effect of immunotherapy, it was speculated that the four ISs may response differently to immunotherapy. For further verification, the tumor response to immune checkpoint inhibitors (ICIs) was evaluated by the Tumor Immune Dysfunction and Exclusion (TIDE) score system (27), and the TIDE score of IS3 patients was found to be the lowest among the four ISs (Figure 5B), suggesting that this LUAD subtype may have a better response to ICIs and also explained the most favorable prognosis of IS3 among the four ISs. In addition, the four subtypes were also significantly correlated with T cell dysfunction score and exclusion score (Figures 5C,D). Considering the fact that chemotherapy is commonly used in cancer treatment, the response of four ISs to commonly used drugs were evaluated. On the basis of predictive model of the four chemo drugs (cisplatin, erlotinib, sorafenib and vinorelbine), the IC_50_ value of each subtype in the TCGA data set was analyzed. Significant differences in the IC_50_ values of all ISs were found in the four chemotherapeutic drugs, and the IS1 with the worst prognosis was more sensitive to four chemo drugs (Figures 5E–H).

### Construction of Immune Cell Signatures-Based Scoring System Through LDA

**Figure 6:**
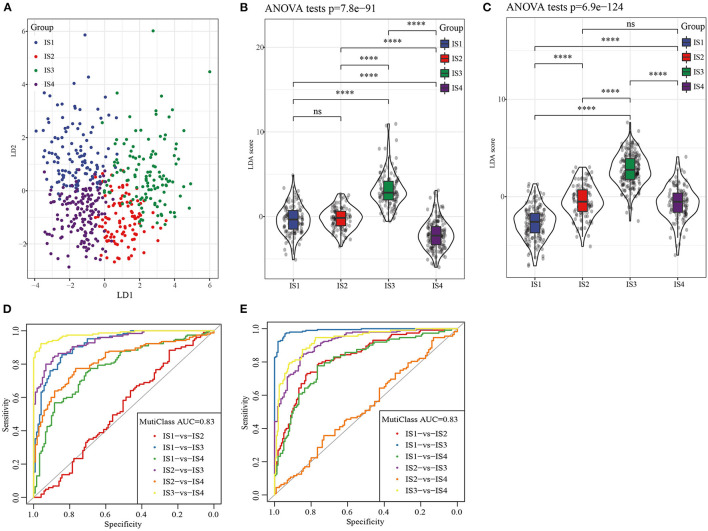
LDA was used to construct a scoring system based on immune cell signatures. **(A)** LDA diagram of four ISs patients in TCGA cohort. **(B)** The violin diagram showed the different LDA score between IS1, IS2, IS3, and IS4 in TCGA. **(C)** Difference analysis of LDA score of four ISs patients in GEO database. **(D)** Receiver operating characteristic (ROC) curves of LDA score in the TCGA. **(E)** Receiver operating characteristic (ROC) curves of LDA score in the GEO.

LDA based on nine immune cell signatures could distinguish different subtypes of LUAD in TCGA (Figure 6A). LDA score of each subtype of LUAD patients in TCGA and GEO database was calculated and differences were analyzed. The results showed that there were significant differences in LAD score among the four subtypes of LUAD patients both in TCGA and GEO databases (Figures 6B,C). According to the results of receiver operating characteristic (ROC) curve analysis, combined area under curve (AUC) of LDA in TCGA was 0.83, similarly combined AUC of LDA in GEO was 0.83 (Figures 6D,E). Therefore, The LDA score model was verified to have a high accuracy in predicting immune characteristics of LUAD.

### The Correlation Between LDA Score and LUAD Immunotherapy Response Was Assessed

**Figure 7:**
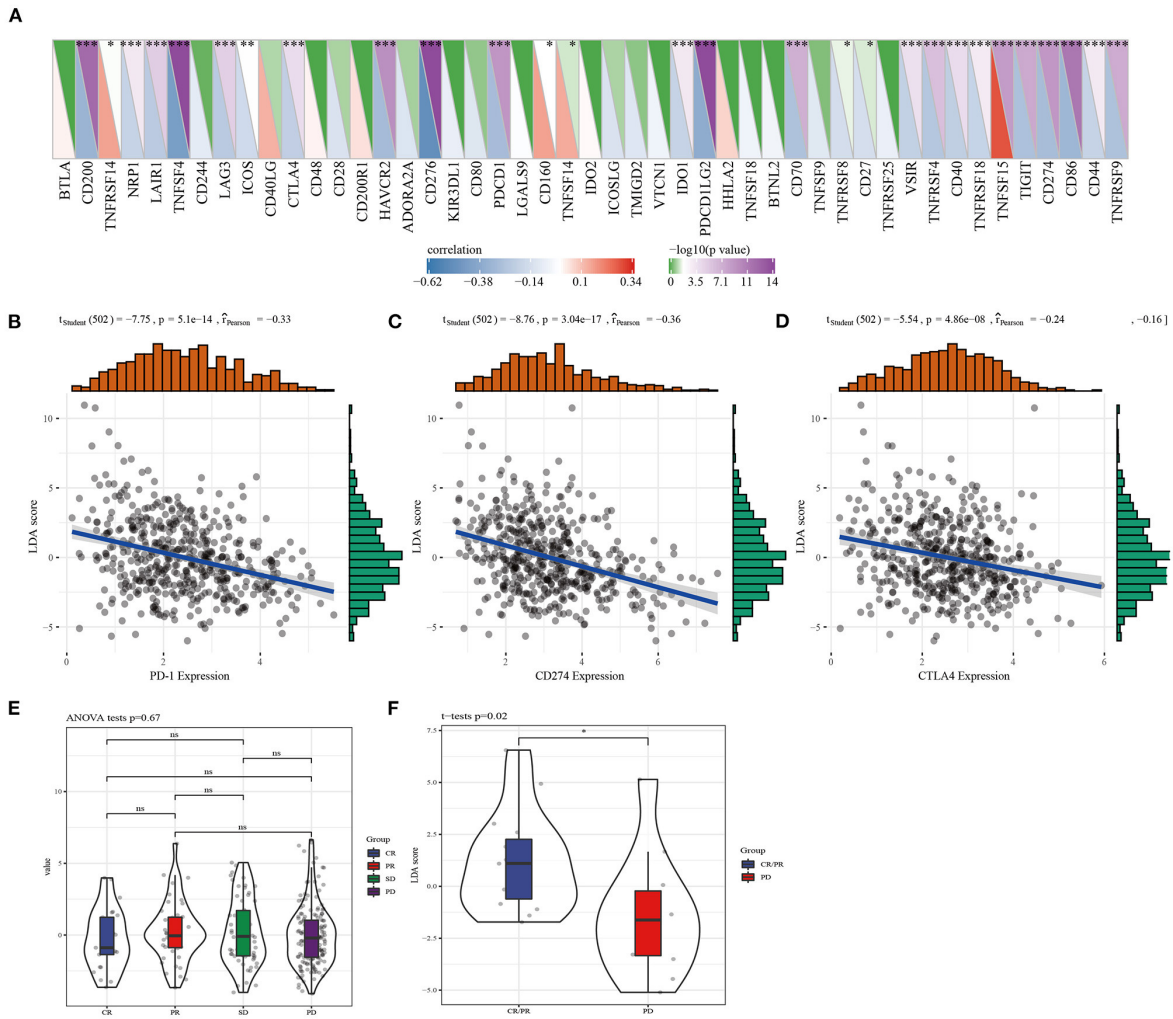
The correlation between LDA score and LUAD immunotherapy response was assessed. **(A)** The immune checkpoints significantly related to LDA score were screened from 47 immune checkpoints. **(B)** Correlation analysis of LDA score and PD1 expression. **(C)** Analysis of correlation between LDA score and CD274 expression. **(D)** Correlation analysis of LDA score and CTLA4 expression. **(E)** LDA score evaluation between CR, PR, SD, and PD before anti-PDL1 treatment. **(F)** The difference of LDA score between CR/PR target lesions and PD target lesions was analyzed.

The correlation between LDA score and immunotherapy response was examined according to the correlation between LDA score and immune checkpoints. We screened 28 immune checkpoints from 47 immune checkpoints, and their expression and LDA score was found to be significantly correlated (Figure 7A). Immune checkpoint blocking of PD-1, PD-L1, and CTLA-4 has emerged as a promising immunotherapy (31). Therefore, correlation analysis was conducted between LDA score and the three immune checkpoint inhibitors, and LDA score was significantly negatively correlated with the expression of PD1 (Figure 7B), PD-L1 (CD274) (Figure 7C), and CTLA-4 (Figure 7D), respectively. In addition, LDA scores under the states of complete response (CR), partial response (PR), stable disease (SD) and progressive disease (PD) were examined based on the expression profile data before anti-PDL1 treatment (32), and no differences were detected (Figure 7E). However, in another anti-PD1 pre-treatment expression profile data (33), the LDA score of CR/PR target lesions was significantly higher than that of PD target lesions (Figure 7F).

### Construction of LUAD Co-expression Module and Identification of Key Modules

**Figure 8:**
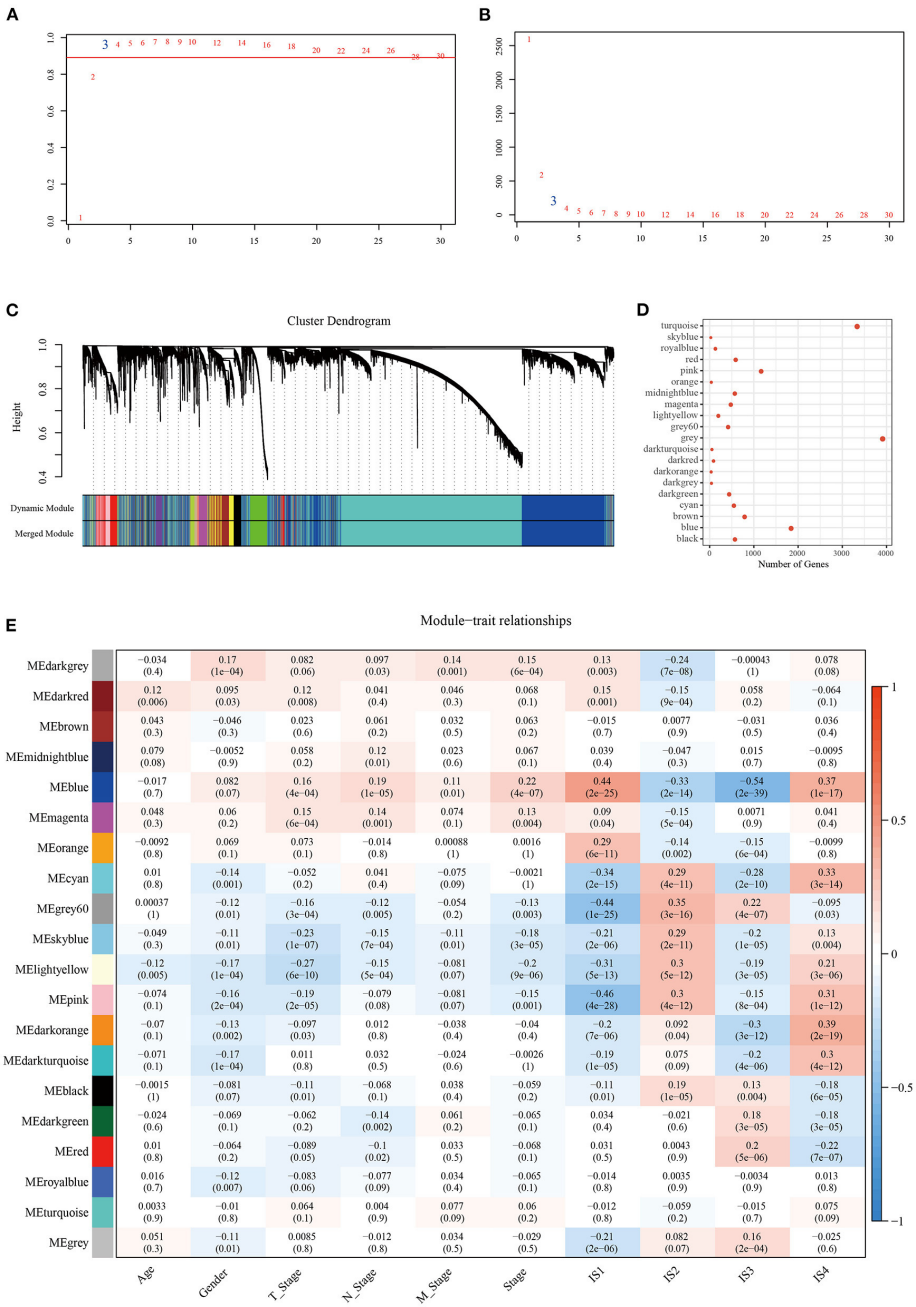
Construction of LUAD co-expression module and identification of key modules. Analysis of **(A)** the scale-free fit index and **(B)** the mean connectivity for various soft-thresholding powers. **(C)** Clustering dendrogram of genes based on topological overlapping. **(D)** A transcript of each module. **(E)** Analysis of module-clinical trait relationships of LUAD.

To identify the modules related to LDA score, firstly, the LUAD samples in TCGA were clustered. The optimal β value in the co-expression network was three, because it was the lowest power with a scale-free topology fitting index greater than 0.90 (Figures 8A,B). Twenty co-expression modules were generated by dynamic tree cutting method (Figure 8C). The transcripts for each module were counted (note that the gray module was a gene module that cannot be assigned) (Figure 8D). To identify ISs-related modules, correlation heatmap between a module and sample traits (age, gender, T stage, N stage, M stage, AJCC stage, IS1, IS2, IS3, IS4) was generated. From the heatmap, it could be observed that the positive correlation between IS1 and blue module was the highest (*r* = 0.44, *p* < 0.05) and the negative correlation with pink module was the strongest (*r* = 0.46, *p* < 0.05), with a significant difference. IS2 showed the highest positive correlation with gray module (*r* = 0.35, *p* < 0.05), and the most significant negative correlation with blue module (*r* = 0.33, *p* < 0.05). Among the 20 modules, IS3 was also the most significantly negatively correlated with blue (*r* = 0.54, *p* < 0.05); IS4 showed the most significant positive correlation with dark orange module (*r* = 0.39, *p* < 0.05) and blue module (*r* = 0.37, *p* < 0.05) (Figure 8E).

### Identification of LUAD Prognostic-Related Modules and Hub Genes

**Figure 9:**
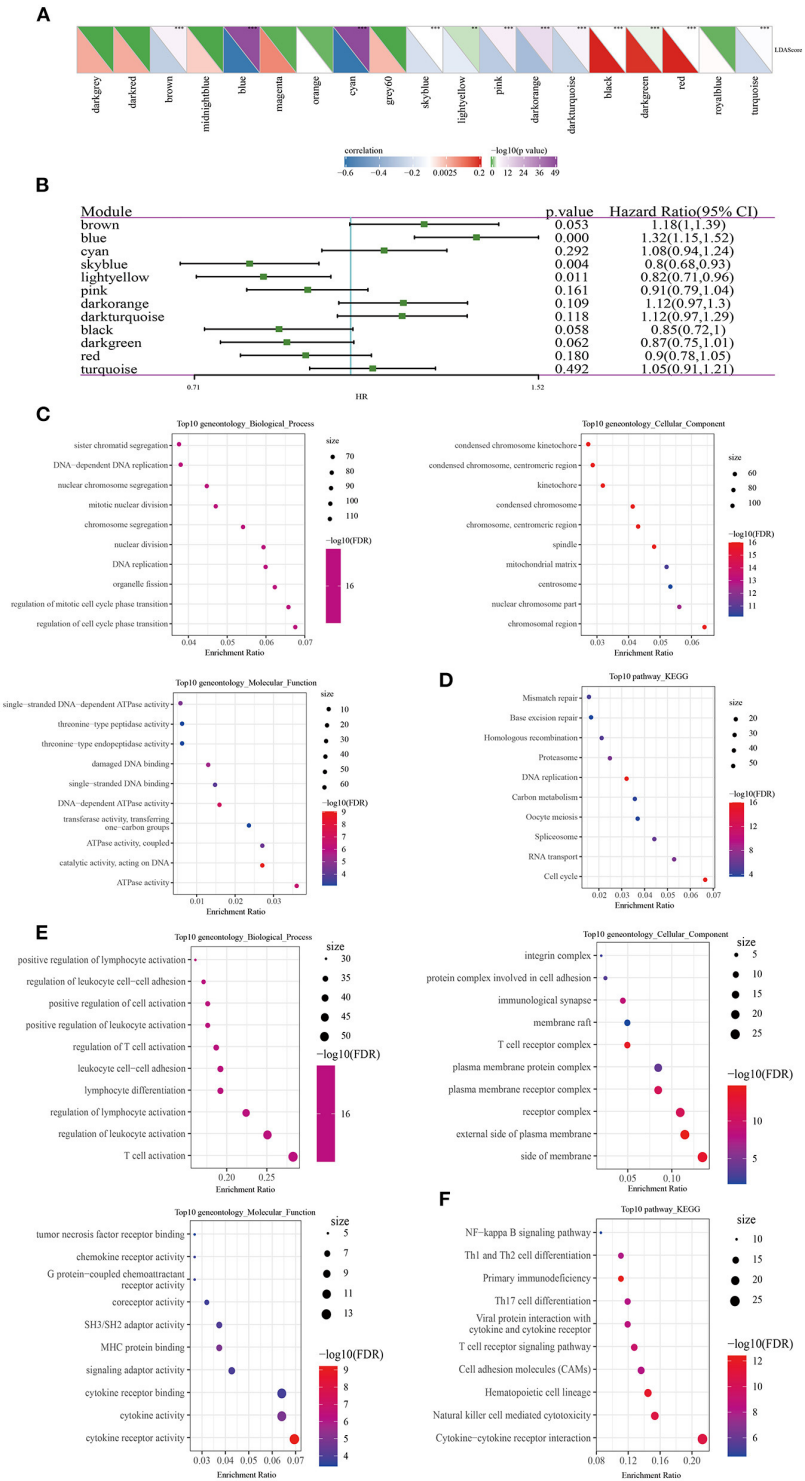
Identification of LUAD prognostic-related modules and hub genes. **(A)** Correlation analysis between modules and LDA score. **(B)** Modules associated with LUAD prognosis were analyzed by univariate Cox screening. **(C)** The top 10 GO terms annotated by blue module annotations. **(D)** The top 10 KEGG pathways annotated by blue module annotations. **(E)** The top 10 GO terms enriched by the light yellow module. **(F)** The top 10 KEGG pathways enriched by the light yellow module.

Correlation analysis determined 12 modules significantly related to LDA score (Figure 9A). Univariate analysis showed that blue module, sky blue module and light yellow were significantly correlated with the prognosis of LUAD (Figure 9B). As the number of genes in the sky blue module was too small, we then focused on the analysis of blue and light yellow modules. In the blue module, hub genes with great prognostic significance were determined to be CCDC90B, ARNTL2, RIPK2, SMCO2, and ADA and NBN (Supplementary Figure 4). A total of 8 hub genes, namely, NLRC3, CLEC2D, GIMAP5, CXorf65, PARP15, AKNA, ZC3H12D, and ARRDC5, were in the light yellow module. Except for CXorf65, the expression of the other seven genes were significantly associated with the prognosis of LUAD (Supplementary Figure 5). To further understand the biological characteristics of each module, we performed functional enrichment analysis on the genes in the blue and the light yellow modules. Figures 9C,D exhibited the top 10 GO terms and the top 10 KEGG pathways with blue module annotation, and genes in the light yellow module were mainly enriched in immune-related pathways (Figures 9E,F).

## Discussion

LUAD is the most common type of lung cancer, accounting for about 40% of all lung cancer cases. According to morphological characteristics, LUAD can be divided into several histological subtypes (34, 35). Among all LUAD, the most general subtype develops via tumorigenesis and progression from atypical adenomatous hyperplasia (AAH) to adenocarcinoma *in situ* (AIS), to minimally invasive adenocarcinoma (MIA), to overt invasive adenocarcinoma with a lepidic pattern (36). Amassed researchers suggested that the WHO LUAD classification should be modified for various patterns to more accurately predict LUAD prognosis (37). At present, histological features are the basis for further subdivision of LUAD into molecular subclasses, and the latest advances in sequencing technology allow LUAD to be classified according to the markers that regulate or influence certain characteristics of the cancer (34). Here, we subdivided LUAD into four molecular subclasses based on 9 immune cell signatures of LUAD, and the results showed significant prognostic differences among the four kinds of ISs patients.

The four types of ISs presented different molecular characteristics, which were reflected in the differences in the number of TMB, mutant genes, chemokines and chemokine receptors. We observed that TP53 mutations were the most common, which was consistent with previous studies (38). A growing body of findings supported the correlation of differential existence of components of the immune system in deciding the evolution of cancer (39). We found that naive B cells, memory B cells, plasma cells, CD8 T cells, memory resting T cells CD4, activated memory T cells CD4 memory, helper follicular T cells, regulatory T cells, resting NK cells, activated NK cells, Monocytes, M0 Macrophages, M1 Macrophages, M2 Macrophages, resting dendritic cells, resting mast cells, activated mast cells and eosinophils displayed notably different scores in the four IS type. To some extent, these findings also reflected the heterogeneity of LUAD. Since the density of most T cells decreases with the progression of the tumor, B cells were related to the prolongation of survival and increase in the late stage, which had a dual effect on tumor recurrence and progression (40). Different immune cell infiltration of the four kinds of ISs may accordingly lead to variations in recurrence and survival of LUAD patients.

Previous studies have found that LUAD subtypes with different molecular and immune characteristics appear different degrees of sensitivity to immunotherapies/chemotherapies (41). Consistently, the current findings showed that the four subtypes responded differently to immune/chemotherapy. It was mainly manifested in the differences in molecular expression of immune checkpoint molecules, TIDE scores, T cell dysfunction scores and exclusion scores among the four kinds of ISs, and the sensitivity to common chemotherapeutic drugs. Although the immune microenvironment of LUAD was comprehensively analyzed, these results may not correctly reflect the inherent characteristics of the tumor, which, however, is also important in regulating the function of immune cells (11). Therefore, we also characterized LUAD by the LDA score of each IS. LDA score was negatively related to the expression of immune checkpoint inhibitors, and also showed differences between CR/PR target lesions and PD target lesions.

More importantly, from 12 modules significantly associated with LDA score, we determined three modules closely associated with the prognosis of LUAD. Hub genes in blue module and light yellow module were screened. A high expression of 6 hub genes in blue module was associated with favorable LUAD prognosis, and they were mainly enriched in cell division-related pathways. In the light yellow module, seven hub genes mainly related to immunity were found to be protective of the survival of LUAD. Notably, most of these hub genes have been identified as prognostic biomarkers or regulators of cancer and were associated with pathologic progression of multiple tumor types. ARNTL2 was a prognostic biomarker of LUAD by promoting multiple organ metastasis and cell proliferation. In addition, high ARNTL2 was a poor prognostic marker for low-grade glioma, renal clear cell carcinoma and pancreatic cancer (42). RIPK2 acts as a tumor marker by regulating NF-κB signaling (43). ADA level in serum may be a biomarker for diagnosis and prognosis of oral squamous cell carcinoma (44). The variant of C. 657DEL5 in the NBN gene increases the risk of pancreatic cancer (45). NLRC3 mediates protection against colorectal cancer by inhibiting the activation of the mTOR signaling pathway (46). CLEC2D expression in lung cancer is linked to better clinical outcomes (47). AKNA is an effective target for diagnosis and treatment since it can regulate EMT-related pathways in gastric cancer (48). The expression of ZC3H12D is closely related to LUAD stage, lymph node metastasis and immune invasion (49). These findings highlight the importance of hub genes in the two modules, which are not independent but represent an important set of LUAD influencing factors for our study. In addition, we also obtained the interaction relationship of these 14 hub genes from the string database. It can be observed that there is less interaction between these genes (Supplementary Figure 6A), suggesting that they may play a role in their respective regulatory pathways. The R software package ClusterProfiler was used to analyze the functional enrichment of 14 hub genes. These genes were mainly enriched in biological processes such as lymphocyte activation involved in immune response, interference alpha production and so on (Supplementary Figure 6B). These hub genes were mainly divided into two parts, and each part of them had several high positive correlations with each other (Supplementary Figure 6C).

Although our study preliminarily explored the immune heterogeneity of different ISs in LUAD through bioinformatics analysis, there were still some limitations. The current sample size was small and came from public database, the population race was mainly limited to whites and blacks, therefore our results should be verified in other races. Moreover, the current research was limited to bioinformatics analysis, and further clinical studies are needed.

## Conclusion

In this study, we identified four LUAD immune subtypes with different molecular characteristics, immune characteristics and prognostic outcomes based on immune cell signatures. In addition, ISs related modules were identified by WGCNA, and LUAD prognostic related modules and 14 hub genes was screened, of which 13 hub genes can be used as potential biomarkers to predict the prognosis of LUAD patients. Our research may provide a potential perspective for immunotherapy.

## Data Availability Statement

The datasets presented in this study can be found in online repositories. The names of the repository/repositories and accession number(s) can be found below: https://portal.gdc.cancer.gov/projects/TCGA-LUAD, https://TCGA-LUAD//www.ncbi.nlm.nih.gov/, GSE37745, GSE19188, GSE50081, GSE30219, GSE31210.

## Author Contributions

LD conceived and designed this study, conducted most of the experiments, data analysis, and wrote the manuscript. YY and GX provided needed funding and resources, and administrated the project. FL, TW, LD, and HC participated in collecting data and helped to draft the manuscript. All authors reviewed and approved the manuscript.

## Funding

This work was financially supported by the National Nature Science Foundation of China (81972025, 81802115) and the Graduate Scientific Research and Innovation Project of Chongqing (CYB20162).

## Conflict of Interest

The authors declare that the research was conducted in the absence of any commercial or financial relationships that could be construed as a potential conflict of interest.

## Publisher's Note

All claims expressed in this article are solely those of the authors and do not necessarily represent those of their affiliated organizations, or those of the publisher, the editors and the reviewers. Any product that may be evaluated in this article, or claim that may be made by its manufacturer, is not guaranteed or endorsed by the publisher.
