# Supplementary figures and images for: Identification of an Immune Classification and Prognostic Genes for Lung Adenocarcinoma Based on Immune Cell Signatures
Source: Front Med (Lausanne). 2022 Mar 30;9:855387. doi: 10.3389/fmed.2022.855387 (PMC9005848; doi:10.3389/fmed.2022.855387)

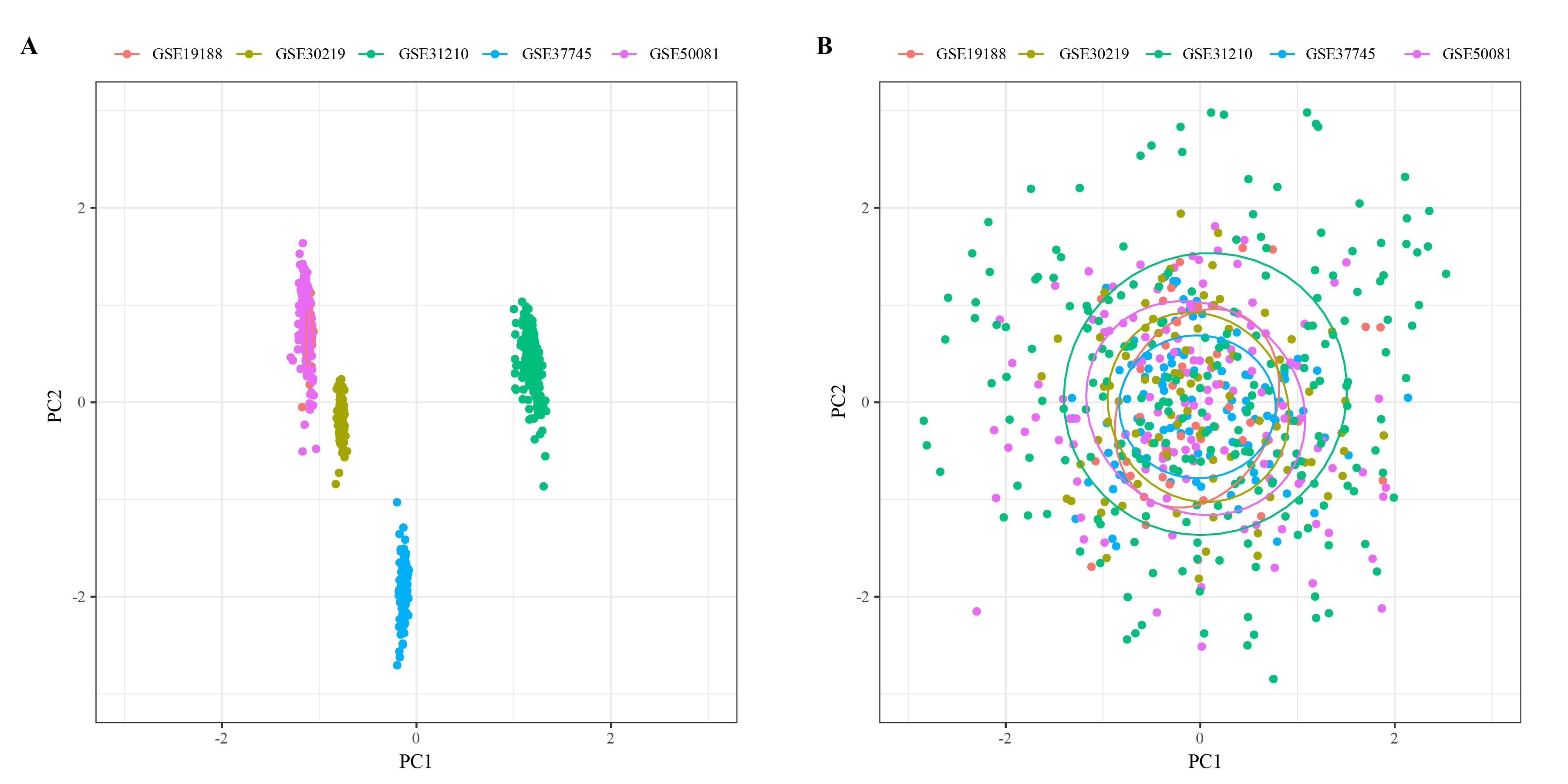

Supplement: Supplementary file 1 [file Image_1.JPEG]

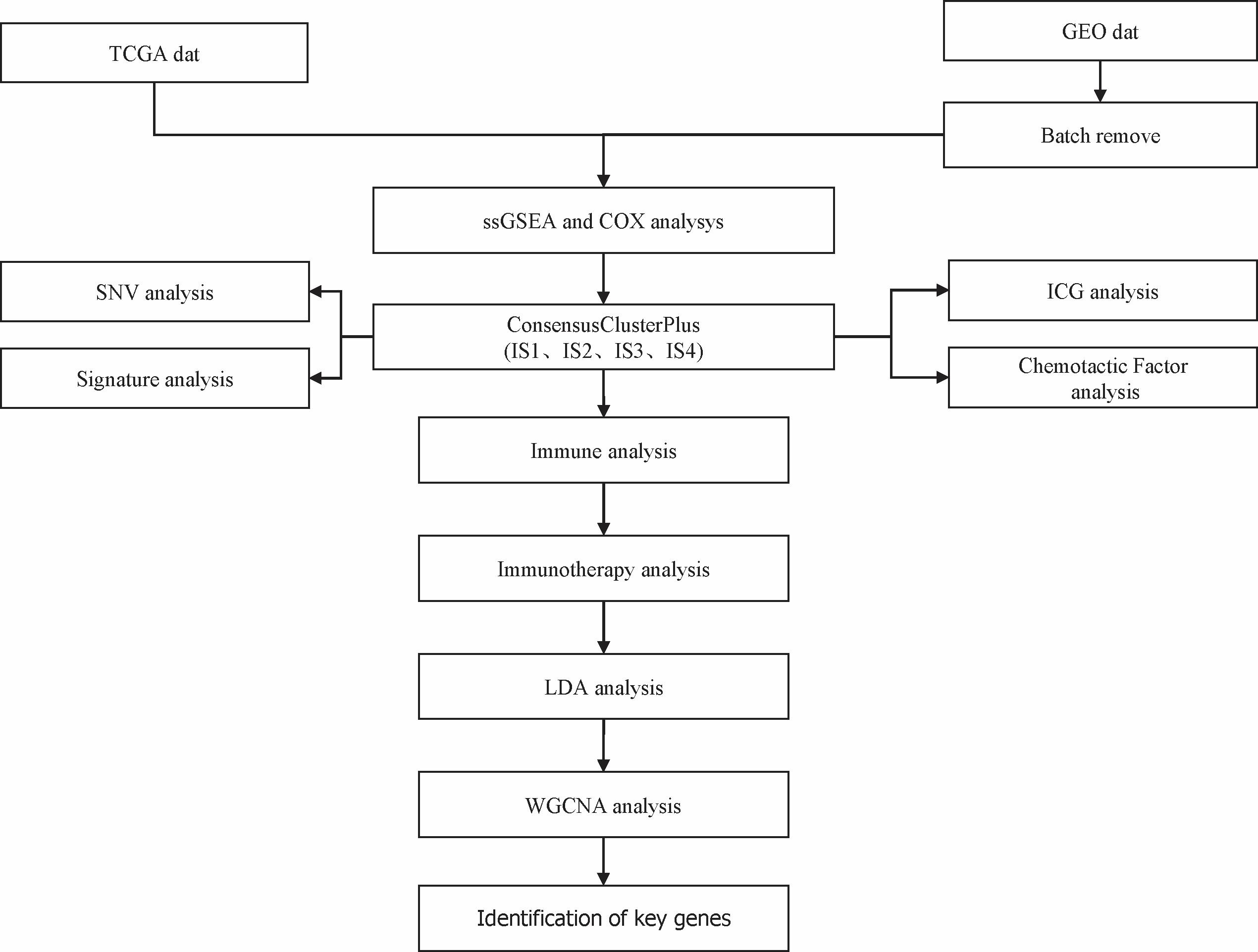

Supplement: Supplementary file 2 [file Image_2.JPEG]

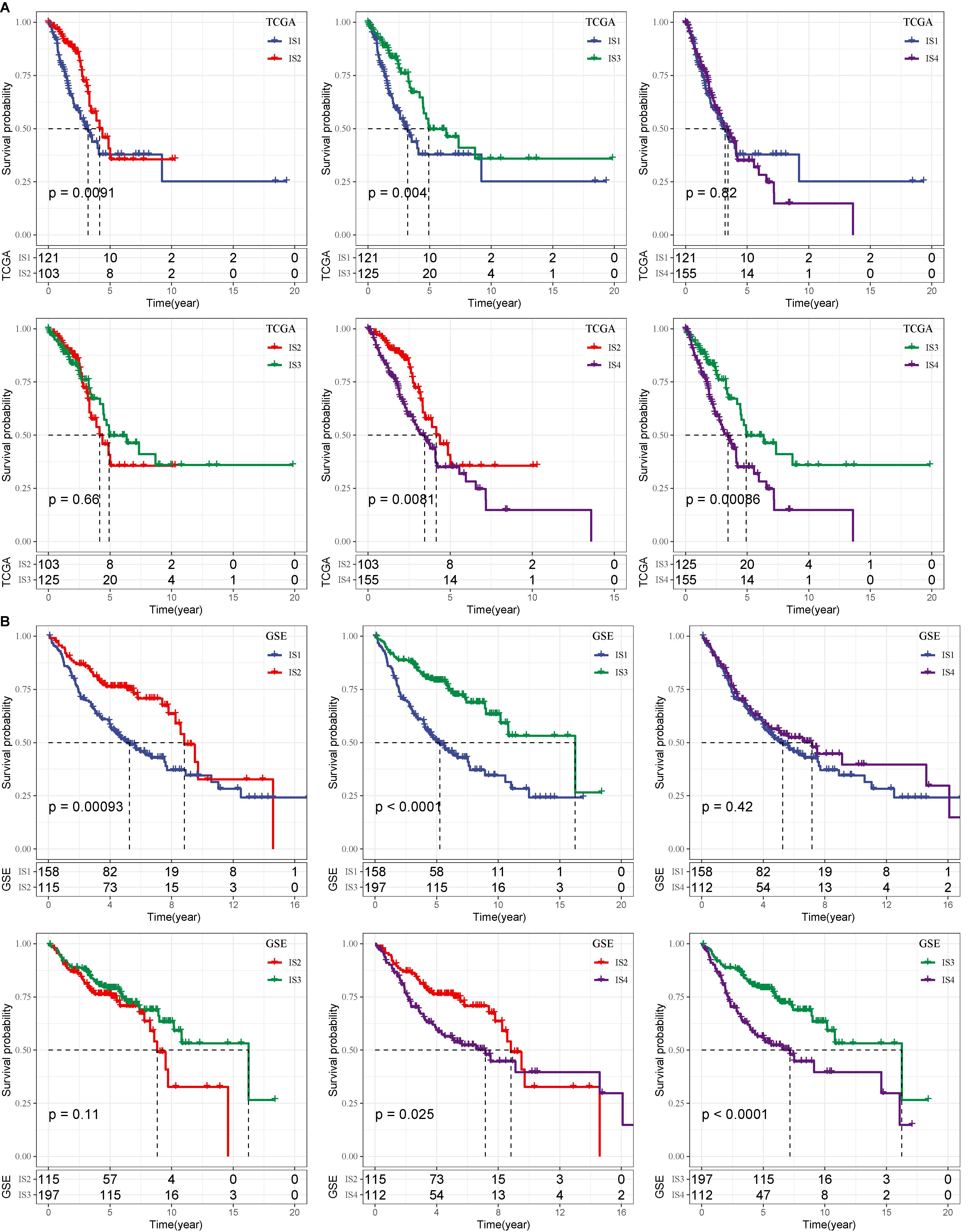

Supplement: Supplementary file 3 [file Image_3.JPEG]

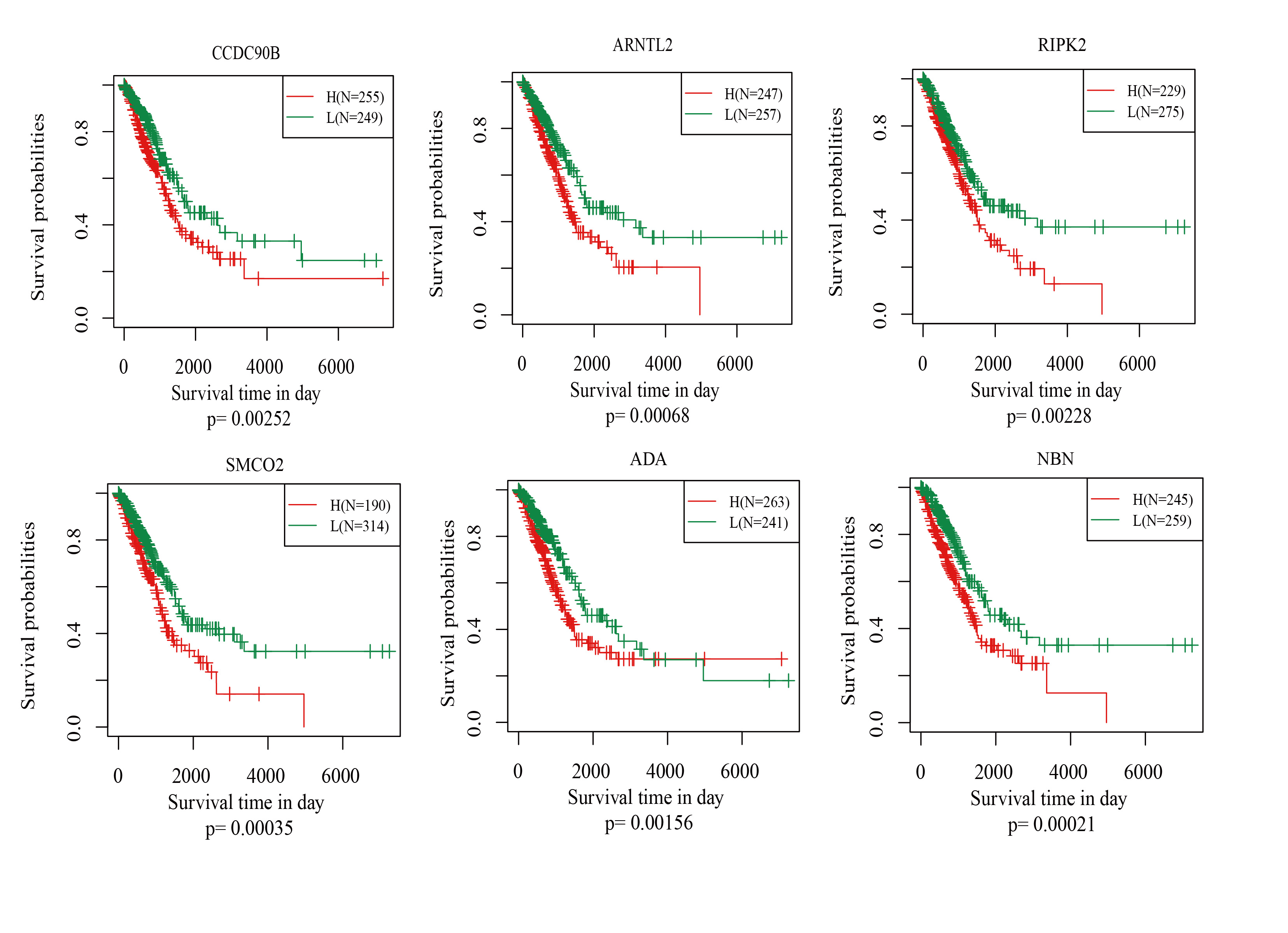

Supplement: Supplementary file 4 [file Image_4.JPEG]

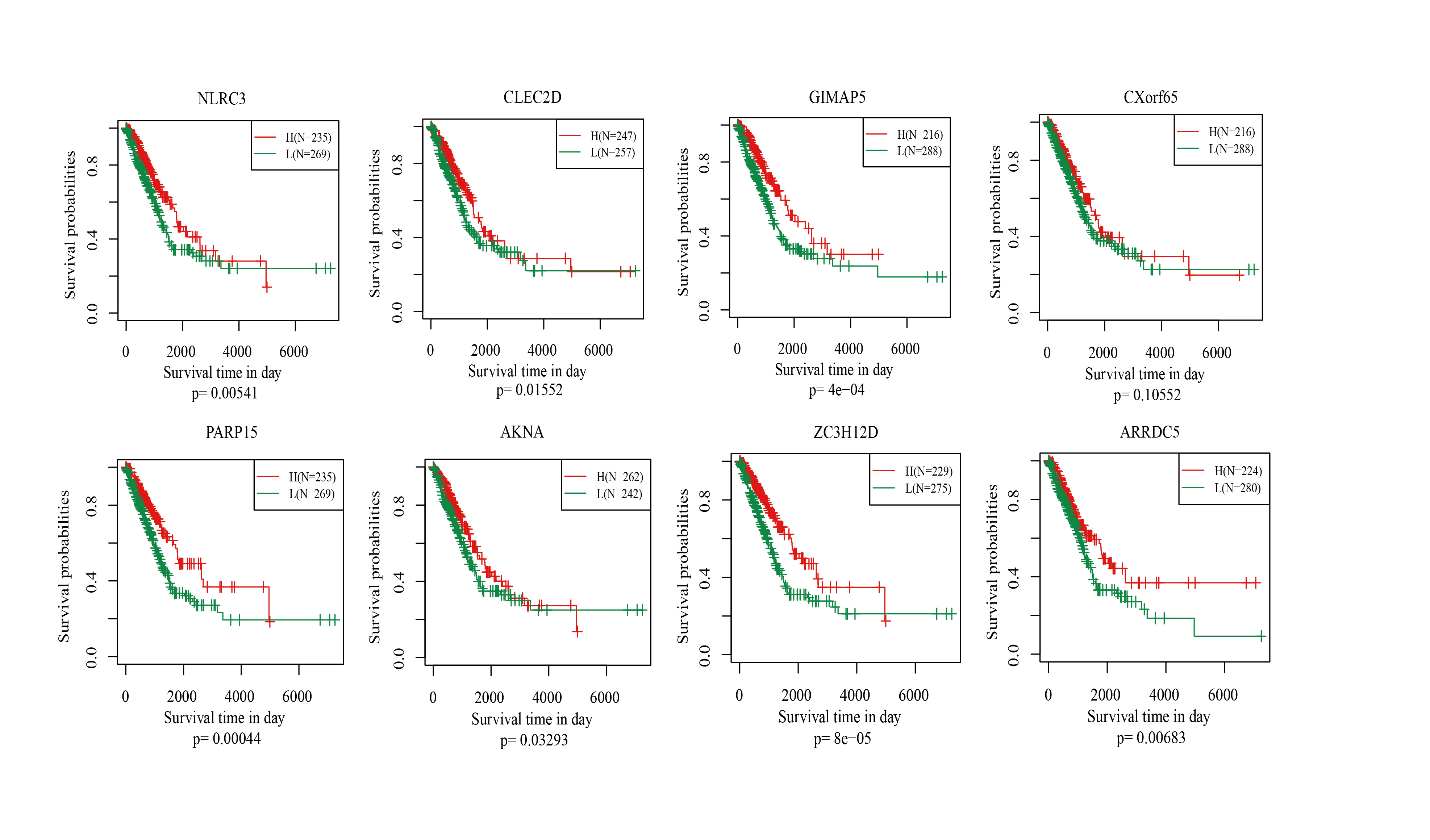

Supplement: Supplementary file 5 [file Image_5.JPEG]

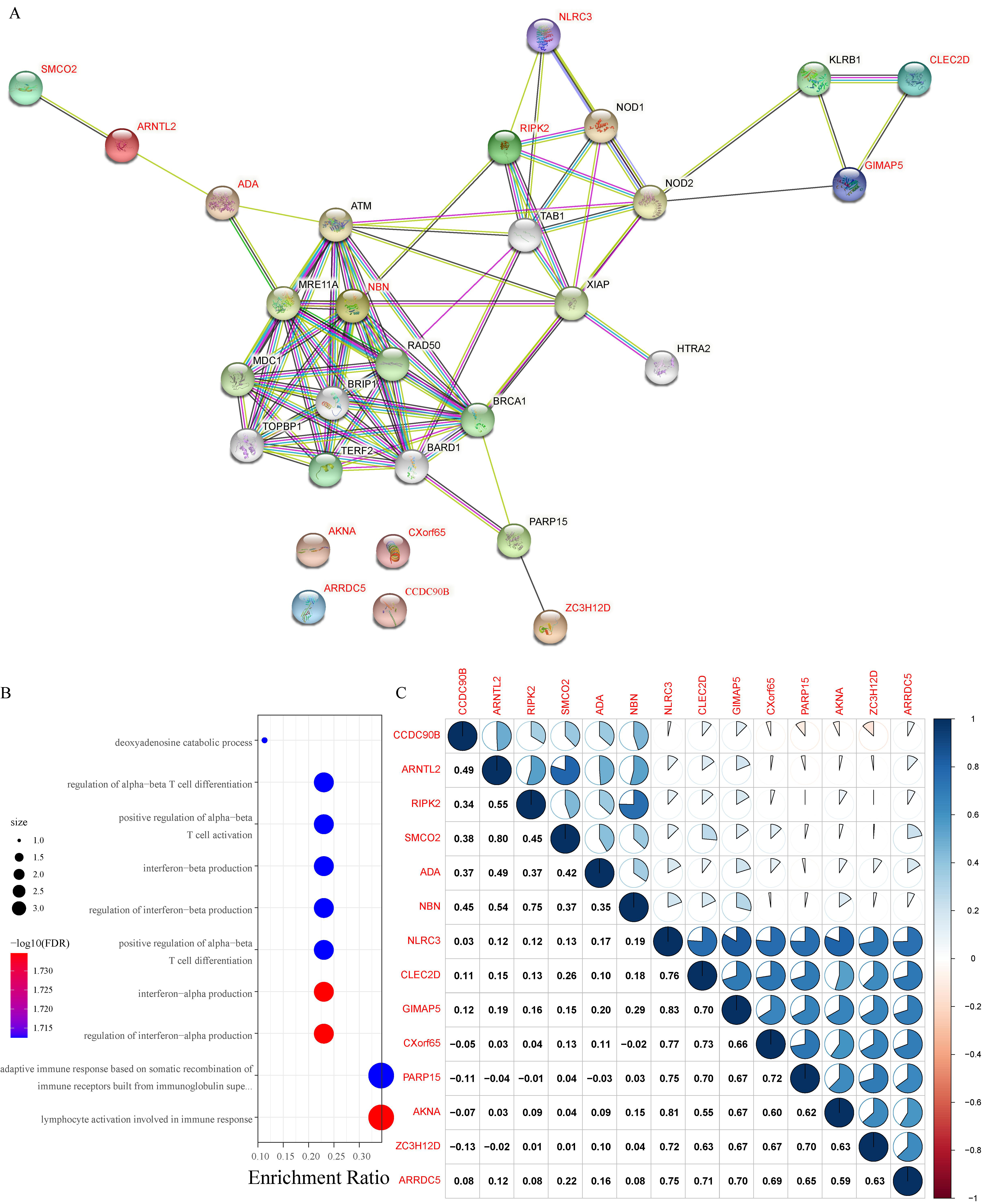

Supplement: Supplementary file 6 [file Image_6.JPEG]
