# Supplementary material for: Identification of an Immune Classification and Prognostic Genes for Lung Adenocarcinoma Based on Immune Cell Signatures
Source: Front Med (Lausanne). 2022 Mar 30;9:855387. doi: 10.3389/fmed.2022.855387 (PMC9005848; doi:10.3389/fmed.2022.855387)
Supplement: Supplementary file 7 [file Table_1.DOCX]

**Supplementary Table 1|**  **Clustering results of samples in TCGA dataset**

| TCGA Sample | Cluster |
| --- | --- |
| TCGA-44-3918-01 | IS1 |
| TCGA-73-4670-01 | IS1 |
| TCGA-86-8280-01 | IS2 |
| TCGA-44-6776-01 | IS3 |
| TCGA-55-A491-01 | IS4 |
| TCGA-44-2668-01 | IS4 |
| TCGA-05-5425-01 | IS4 |
| TCGA-78-8662-01 | IS1 |
| TCGA-S2-AA1A-01 | IS2 |
| TCGA-55-A494-01 | IS1 |
| TCGA-50-6592-01 | IS4 |
| TCGA-95-8039-01 | IS2 |
| TCGA-50-8459-01 | IS2 |
| TCGA-55-8507-01 | IS1 |
| TCGA-50-5939-01 | IS4 |
| TCGA-49-AARO-01 | IS2 |
| TCGA-38-4631-01 | IS1 |
| TCGA-NJ-A55R-01 | IS1 |
| TCGA-53-7624-01 | IS1 |
| TCGA-38-4630-01 | IS1 |
| TCGA-64-5778-01 | IS4 |
| TCGA-MP-A4TI-01 | IS4 |
| TCGA-97-8176-01 | IS1 |
| TCGA-55-6981-01 | IS4 |
| TCGA-55-8299-01 | IS4 |
| TCGA-55-A4DG-01 | IS3 |
| TCGA-55-8087-01 | IS3 |
| TCGA-97-7546-01 | IS2 |
| TCGA-86-6562-01 | IS4 |
| TCGA-95-7947-01 | IS1 |
| TCGA-97-A4LX-01 | IS2 |
| TCGA-05-4415-01 | IS1 |
| TCGA-49-4488-01 | IS3 |
| TCGA-62-A470-01 | IS3 |
| TCGA-05-4432-01 | IS4 |
| TCGA-86-8674-01 | IS3 |
| TCGA-MP-A4SV-01 | IS4 |
| TCGA-78-7536-01 | IS1 |
| TCGA-78-7159-01 | IS4 |
| TCGA-95-7039-01 | IS4 |
| TCGA-38-7271-01 | IS2 |
| TCGA-44-2662-01 | IS1 |
| TCGA-05-5429-01 | IS1 |
| TCGA-99-AA5R-01 | IS2 |
| TCGA-55-6984-01 | IS1 |
| TCGA-93-A4JQ-01 | IS4 |
| TCGA-55-7816-01 | IS2 |
| TCGA-55-A48X-01 | IS2 |
| TCGA-97-A4M1-01 | IS3 |
| TCGA-J2-8192-01 | IS2 |
| TCGA-91-6836-01 | IS1 |
| TCGA-49-4494-01 | IS4 |
| TCGA-55-A48Y-01 | IS4 |
| TCGA-97-7937-01 | IS1 |
| TCGA-44-A47A-01 | IS2 |
| TCGA-44-6775-01 | IS3 |
| TCGA-86-A4P8-01 | IS3 |
| TCGA-64-1679-01 | IS4 |
| TCGA-55-8085-01 | IS4 |
| TCGA-55-A490-01 | IS4 |
| TCGA-64-1680-01 | IS3 |
| TCGA-49-4514-01 | IS1 |
| TCGA-55-7994-01 | IS1 |
| TCGA-44-2666-01 | IS3 |
| TCGA-44-2665-01 | IS2 |
| TCGA-50-6590-01 | IS4 |
| TCGA-MP-A5C7-01 | IS3 |
| TCGA-67-6216-01 | IS2 |
| TCGA-50-5936-01 | IS4 |
| TCGA-75-7027-01 | IS4 |
| TCGA-97-8172-01 | IS3 |
| TCGA-44-8119-01 | IS4 |
| TCGA-80-5611-01 | IS1 |
| TCGA-69-7979-01 | IS1 |
| TCGA-38-4628-01 | IS1 |
| TCGA-55-8206-01 | IS3 |
| TCGA-55-A4DF-01 | IS1 |
| TCGA-38-A44F-01 | IS2 |
| TCGA-55-6975-01 | IS4 |
| TCGA-35-4122-01 | IS4 |
| TCGA-44-7669-01 | IS1 |
| TCGA-35-4123-01 | IS4 |
| TCGA-86-8671-01 | IS2 |
| TCGA-44-A4SS-01 | IS4 |
| TCGA-05-4244-01 | IS4 |
| TCGA-NJ-A7XG-01 | IS3 |
| TCGA-05-4384-01 | IS3 |
| TCGA-62-8397-01 | IS3 |
| TCGA-64-1678-01 | IS1 |
| TCGA-91-8499-01 | IS1 |
| TCGA-44-5644-01 | IS1 |
| TCGA-50-5935-01 | IS3 |
| TCGA-99-8025-01 | IS2 |
| TCGA-55-8301-01 | IS4 |
| TCGA-78-7150-01 | IS4 |
| TCGA-MP-A4T6-01 | IS3 |
| TCGA-69-7761-01 | IS4 |
| TCGA-55-8203-01 | IS3 |
| TCGA-50-5930-01 | IS4 |
| TCGA-50-5933-01 | IS4 |
| TCGA-55-8512-01 | IS3 |
| TCGA-83-5908-01 | IS4 |
| TCGA-NJ-A55A-01 | IS3 |
| TCGA-69-7765-01 | IS2 |
| TCGA-86-8076-01 | IS2 |
| TCGA-93-7348-01 | IS2 |
| TCGA-97-7552-01 | IS3 |
| TCGA-05-4427-01 | IS4 |
| TCGA-55-8094-01 | IS1 |
| TCGA-44-A47B-01 | IS3 |
| TCGA-L4-A4E5-01 | IS1 |
| TCGA-55-7725-01 | IS3 |
| TCGA-71-6725-01 | IS1 |
| TCGA-73-4675-01 | IS3 |
| TCGA-44-3917-01 | IS1 |
| TCGA-73-4676-01 | IS1 |
| TCGA-86-8054-01 | IS1 |
| TCGA-69-7764-01 | IS3 |
| TCGA-50-5068-01 | IS4 |
| TCGA-55-6712-01 | IS4 |
| TCGA-44-2656-01 | IS4 |
| TCGA-55-5899-01 | IS1 |
| TCGA-44-6778-01 | IS4 |
| TCGA-78-7220-01 | IS1 |
| TCGA-05-4382-01 | IS4 |
| TCGA-95-7567-01 | IS4 |
| TCGA-64-1676-01 | IS4 |
| TCGA-97-8174-01 | IS3 |
| TCGA-55-8090-01 | IS2 |
| TCGA-55-7903-01 | IS1 |
| TCGA-49-AAQV-01 | IS4 |
| TCGA-55-7995-01 | IS4 |
| TCGA-55-7281-01 | IS2 |
| TCGA-91-6835-01 | IS4 |
| TCGA-49-6767-01 | IS4 |
| TCGA-05-4389-01 | IS4 |
| TCGA-44-4112-01 | IS4 |
| TCGA-55-7914-01 | IS1 |
| TCGA-MP-A4TJ-01 | IS2 |
| TCGA-69-7980-01 | IS2 |
| TCGA-78-7145-01 | IS4 |
| TCGA-67-6215-01 | IS3 |
| TCGA-L9-A50W-01 | IS3 |
| TCGA-95-7043-01 | IS1 |
| TCGA-MP-A4TA-01 | IS1 |
| TCGA-97-7547-01 | IS3 |
| TCGA-97-7938-01 | IS3 |
| TCGA-J2-A4AD-01 | IS1 |
| TCGA-44-7670-01 | IS1 |
| TCGA-L9-A5IP-01 | IS1 |
| TCGA-49-6744-01 | IS2 |
| TCGA-55-6642-01 | IS4 |
| TCGA-50-5051-01 | IS1 |
| TCGA-55-8205-01 | IS4 |
| TCGA-55-8508-01 | IS2 |
| TCGA-44-2657-01 | IS2 |
| TCGA-44-6145-01 | IS4 |
| TCGA-91-6831-01 | IS1 |
| TCGA-55-8620-01 | IS1 |
| TCGA-49-AARR-01 | IS3 |
| TCGA-50-7109-01 | IS1 |
| TCGA-55-8614-01 | IS4 |
| TCGA-50-5931-01 | IS1 |
| TCGA-64-5775-01 | IS4 |
| TCGA-05-4249-01 | IS2 |
| TCGA-O1-A52J-01 | IS3 |
| TCGA-97-7554-01 | IS2 |
| TCGA-05-4402-01 | IS4 |
| TCGA-69-A59K-01 | IS1 |
| TCGA-44-6147-01 | IS2 |
| TCGA-MP-A4SW-01 | IS2 |
| TCGA-91-6847-01 | IS1 |
| TCGA-95-7562-01 | IS1 |
| TCGA-62-A46R-01 | IS3 |
| TCGA-MP-A4TC-01 | IS4 |
| TCGA-97-8177-01 | IS2 |
| TCGA-67-3770-01 | IS3 |
| TCGA-49-4505-01 | IS2 |
| TCGA-L9-A743-01 | IS4 |
| TCGA-86-8358-01 | IS1 |
| TCGA-62-A471-01 | IS1 |
| TCGA-05-4250-01 | IS4 |
| TCGA-55-8208-01 | IS4 |
| TCGA-62-8402-01 | IS4 |
| TCGA-93-A4JP-01 | IS2 |
| TCGA-67-3772-01 | IS2 |
| TCGA-44-5645-01 | IS3 |
| TCGA-55-7570-01 | IS1 |
| TCGA-44-6146-01 | IS3 |
| TCGA-44-7662-01 | IS4 |
| TCGA-55-8510-01 | IS2 |
| TCGA-NJ-A4YG-01 | IS2 |
| TCGA-78-7158-01 | IS3 |
| TCGA-55-7815-01 | IS4 |
| TCGA-93-8067-01 | IS4 |
| TCGA-99-8033-01 | IS4 |
| TCGA-91-A4BC-01 | IS4 |
| TCGA-38-6178-01 | IS2 |
| TCGA-78-8640-01 | IS1 |
| TCGA-44-3398-01 | IS2 |
| TCGA-99-8032-01 | IS4 |
| TCGA-49-AAR2-01 | IS3 |
| TCGA-78-8655-01 | IS3 |
| TCGA-78-7535-01 | IS1 |
| TCGA-44-6777-01 | IS2 |
| TCGA-55-6972-01 | IS3 |
| TCGA-49-6742-01 | IS1 |
| TCGA-86-8056-01 | IS3 |
| TCGA-69-7978-01 | IS4 |
| TCGA-95-7948-01 | IS3 |
| TCGA-55-6979-01 | IS4 |
| TCGA-55-7284-01 | IS3 |
| TCGA-78-7542-01 | IS1 |
| TCGA-55-8514-01 | IS3 |
| TCGA-NJ-A4YI-01 | IS3 |
| TCGA-97-A4M0-01 | IS3 |
| TCGA-49-4506-01 | IS4 |
| TCGA-55-A493-01 | IS4 |
| TCGA-78-7156-01 | IS3 |
| TCGA-44-2659-01 | IS3 |
| TCGA-78-7143-01 | IS4 |
| TCGA-53-7626-01 | IS2 |
| TCGA-55-7726-01 | IS4 |
| TCGA-55-8619-01 | IS3 |
| TCGA-95-7944-01 | IS1 |
| TCGA-86-7953-01 | IS4 |
| TCGA-J2-8194-01 | IS3 |
| TCGA-62-8394-01 | IS4 |
| TCGA-69-8255-01 | IS1 |
| TCGA-MP-A4T7-01 | IS4 |
| TCGA-44-7660-01 | IS1 |
| TCGA-55-7573-01 | IS3 |
| TCGA-99-8028-01 | IS2 |
| TCGA-86-6851-01 | IS2 |
| TCGA-MP-A4SY-01 | IS4 |
| TCGA-97-A4M5-01 | IS3 |
| TCGA-86-8073-01 | IS2 |
| TCGA-78-8660-01 | IS4 |
| TCGA-49-6743-01 | IS1 |
| TCGA-49-6761-01 | IS4 |
| TCGA-75-6212-01 | IS2 |
| TCGA-05-4417-01 | IS2 |
| TCGA-78-7166-01 | IS1 |
| TCGA-05-5428-01 | IS1 |
| TCGA-50-5944-01 | IS2 |
| TCGA-69-8254-01 | IS3 |
| TCGA-44-5643-01 | IS4 |
| TCGA-97-A4M7-01 | IS2 |
| TCGA-05-4426-01 | IS2 |
| TCGA-55-8089-01 | IS4 |
| TCGA-50-5066-01 | IS4 |
| TCGA-91-A4BD-01 | IS3 |
| TCGA-55-8207-01 | IS3 |
| TCGA-05-4433-01 | IS3 |
| TCGA-55-A48Z-01 | IS4 |
| TCGA-75-5146-01 | IS3 |
| TCGA-62-A46Y-01 | IS3 |
| TCGA-78-7539-01 | IS3 |
| TCGA-53-7813-01 | IS1 |
| TCGA-75-7025-01 | IS3 |
| TCGA-MP-A4T9-01 | IS2 |
| TCGA-67-3771-01 | IS4 |
| TCGA-67-6217-01 | IS3 |
| TCGA-49-4507-01 | IS1 |
| TCGA-55-7283-01 | IS2 |
| TCGA-05-4418-01 | IS1 |
| TCGA-55-7576-01 | IS4 |
| TCGA-49-4487-01 | IS4 |
| TCGA-L9-A8F4-01 | IS4 |
| TCGA-L9-A443-01 | IS2 |
| TCGA-73-4677-01 | IS3 |
| TCGA-50-6591-01 | IS1 |
| TCGA-97-A4M3-01 | IS3 |
| TCGA-91-6848-01 | IS4 |
| TCGA-55-1596-01 | IS1 |
| TCGA-97-A4M6-01 | IS2 |
| TCGA-49-4486-01 | IS3 |
| TCGA-73-A9RS-01 | IS1 |
| TCGA-55-8511-01 | IS4 |
| TCGA-L9-A444-01 | IS2 |
| TCGA-91-6840-01 | IS1 |
| TCGA-95-A4VK-01 | IS3 |
| TCGA-86-8075-01 | IS4 |
| TCGA-55-6986-01 | IS3 |
| TCGA-73-7499-01 | IS4 |
| TCGA-55-8096-01 | IS2 |
| TCGA-05-4405-01 | IS2 |
| TCGA-64-5779-01 | IS4 |
| TCGA-50-6593-01 | IS4 |
| TCGA-64-1677-01 | IS1 |
| TCGA-55-6983-01 | IS2 |
| TCGA-93-7347-01 | IS2 |
| TCGA-05-4434-01 | IS4 |
| TCGA-91-6849-01 | IS3 |
| TCGA-62-A46S-01 | IS3 |
| TCGA-05-4395-01 | IS1 |
| TCGA-97-7553-01 | IS2 |
| TCGA-50-5941-01 | IS4 |
| TCGA-05-5423-01 | IS3 |
| TCGA-62-8398-01 | IS4 |
| TCGA-MP-A4TK-01 | IS4 |
| TCGA-91-8497-01 | IS3 |
| TCGA-50-6594-01 | IS4 |
| TCGA-44-8117-01 | IS1 |
| TCGA-4B-A93V-01 | IS1 |
| TCGA-55-7910-01 | IS1 |
| TCGA-MN-A4N1-01 | IS1 |
| TCGA-44-7671-01 | IS2 |
| TCGA-44-7659-01 | IS3 |
| TCGA-64-5774-01 | IS1 |
| TCGA-55-A492-01 | IS3 |
| TCGA-86-A4D0-01 | IS1 |
| TCGA-95-8494-01 | IS1 |
| TCGA-38-4632-01 | IS4 |
| TCGA-35-5375-01 | IS1 |
| TCGA-05-4396-01 | IS3 |
| TCGA-J2-A4AG-01 | IS2 |
| TCGA-55-8615-01 | IS1 |
| TCGA-55-7728-01 | IS3 |
| TCGA-97-8552-01 | IS3 |
| TCGA-44-2655-01 | IS3 |
| TCGA-50-6597-01 | IS3 |
| TCGA-55-7727-01 | IS4 |
| TCGA-55-6982-01 | IS4 |
| TCGA-55-6985-01 | IS4 |
| TCGA-MN-A4N4-01 | IS2 |
| TCGA-67-3773-01 | IS3 |
| TCGA-86-A4P7-01 | IS2 |
| TCGA-86-8669-01 | IS3 |
| TCGA-49-4510-01 | IS3 |
| TCGA-49-AARN-01 | IS2 |
| TCGA-50-6595-01 | IS4 |
| TCGA-75-5125-01 | IS4 |
| TCGA-99-7458-01 | IS2 |
| TCGA-49-AAR4-01 | IS4 |
| TCGA-55-6978-01 | IS4 |
| TCGA-55-8204-01 | IS4 |
| TCGA-97-8171-01 | IS3 |
| TCGA-86-7713-01 | IS1 |
| TCGA-55-6968-01 | IS1 |
| TCGA-35-3615-01 | IS3 |
| TCGA-78-7155-01 | IS1 |
| TCGA-50-8460-01 | IS3 |
| TCGA-86-7955-01 | IS1 |
| TCGA-64-5781-01 | IS4 |
| TCGA-78-7154-01 | IS1 |
| TCGA-44-6779-01 | IS4 |
| TCGA-50-5055-01 | IS2 |
| TCGA-55-6980-01 | IS2 |
| TCGA-50-5049-01 | IS4 |
| TCGA-NJ-A4YP-01 | IS1 |
| TCGA-73-4658-01 | IS2 |
| TCGA-05-5715-01 | IS3 |
| TCGA-69-7763-01 | IS2 |
| TCGA-91-7771-01 | IS2 |
| TCGA-05-4403-01 | IS3 |
| TCGA-71-8520-01 | IS4 |
| TCGA-86-8281-01 | IS3 |
| TCGA-78-7160-01 | IS4 |
| TCGA-55-7913-01 | IS1 |
| TCGA-55-7574-01 | IS2 |
| TCGA-L9-A7SV-01 | IS1 |
| TCGA-MP-A4TF-01 | IS1 |
| TCGA-62-A46O-01 | IS1 |
| TCGA-44-A47G-01 | IS2 |
| TCGA-05-4390-01 | IS1 |
| TCGA-91-8496-01 | IS3 |
| TCGA-62-8395-01 | IS3 |
| TCGA-69-8453-01 | IS2 |
| TCGA-93-A4JO-01 | IS2 |
| TCGA-55-8092-01 | IS1 |
| TCGA-55-1592-01 | IS1 |
| TCGA-38-4629-01 | IS4 |
| TCGA-69-7760-01 | IS1 |
| TCGA-44-7672-01 | IS4 |
| TCGA-44-6148-01 | IS3 |
| TCGA-64-5815-01 | IS4 |
| TCGA-97-A4M2-01 | IS3 |
| TCGA-NJ-A4YF-01 | IS1 |
| TCGA-55-1594-01 | IS1 |
| TCGA-75-6206-01 | IS3 |
| TCGA-44-7667-01 | IS1 |
| TCGA-55-7724-01 | IS4 |
| TCGA-55-8506-01 | IS1 |
| TCGA-86-8359-01 | IS2 |
| TCGA-05-4397-01 | IS1 |
| TCGA-75-6214-01 | IS4 |
| TCGA-86-8074-01 | IS4 |
| TCGA-62-A472-01 | IS4 |
| TCGA-78-7149-01 | IS3 |
| TCGA-55-A57B-01 | IS3 |
| TCGA-67-3774-01 | IS2 |
| TCGA-05-4425-01 | IS2 |
| TCGA-55-8505-01 | IS1 |
| TCGA-86-8279-01 | IS4 |
| TCGA-62-8399-01 | IS1 |
| TCGA-78-7633-01 | IS3 |
| TCGA-05-4430-01 | IS2 |
| TCGA-97-8179-01 | IS3 |
| TCGA-05-4410-01 | IS2 |
| TCGA-05-4420-01 | IS1 |
| TCGA-78-7147-01 | IS1 |
| TCGA-50-8457-01 | IS2 |
| TCGA-78-7162-01 | IS3 |
| TCGA-NJ-A55O-01 | IS3 |
| TCGA-50-5044-01 | IS4 |
| TCGA-93-A4JN-01 | IS4 |
| TCGA-91-6829-01 | IS4 |
| TCGA-69-8253-01 | IS3 |
| TCGA-97-8175-01 | IS4 |
| TCGA-55-8097-01 | IS3 |
| TCGA-44-3919-01 | IS2 |
| TCGA-86-8055-01 | IS4 |
| TCGA-78-7161-01 | IS3 |
| TCGA-J2-A4AE-01 | IS3 |
| TCGA-44-2661-01 | IS2 |
| TCGA-05-5420-01 | IS4 |
| TCGA-MP-A4TH-01 | IS3 |
| TCGA-L4-A4E6-01 | IS3 |
| TCGA-50-5946-01 | IS1 |
| TCGA-97-8547-01 | IS2 |
| TCGA-86-7954-01 | IS4 |
| TCGA-78-8648-01 | IS2 |
| TCGA-38-4625-01 | IS1 |
| TCGA-55-8513-01 | IS3 |
| TCGA-38-4626-01 | IS2 |
| TCGA-91-6830-01 | IS4 |
| TCGA-44-7661-01 | IS4 |
| TCGA-73-4668-01 | IS4 |
| TCGA-49-AAR3-01 | IS4 |
| TCGA-50-5932-01 | IS3 |
| TCGA-86-7714-01 | IS3 |
| TCGA-97-7941-01 | IS3 |
| TCGA-55-7907-01 | IS4 |
| TCGA-64-1681-01 | IS2 |
| TCGA-38-4627-01 | IS4 |
| TCGA-86-7711-01 | IS4 |
| TCGA-49-4501-01 | IS2 |
| TCGA-44-6774-01 | IS4 |
| TCGA-78-7152-01 | IS2 |
| TCGA-49-4490-01 | IS2 |
| TCGA-55-7911-01 | IS1 |
| TCGA-78-7167-01 | IS3 |
| TCGA-86-8673-01 | IS1 |
| TCGA-MP-A4TE-01 | IS1 |
| TCGA-44-8120-01 | IS2 |
| TCGA-NJ-A4YQ-01 | IS1 |
| TCGA-MN-A4N5-01 | IS1 |
| TCGA-05-4398-01 | IS4 |
| TCGA-78-7153-01 | IS3 |
| TCGA-50-5942-01 | IS3 |
| TCGA-73-4662-01 | IS2 |
| TCGA-05-4422-01 | IS3 |
| TCGA-75-5147-01 | IS4 |
| TCGA-50-6673-01 | IS2 |
| TCGA-55-7227-01 | IS2 |
| TCGA-73-7498-01 | IS3 |
| TCGA-69-7973-01 | IS1 |
| TCGA-53-A4EZ-01 | IS1 |
| TCGA-80-5608-01 | IS1 |
| TCGA-49-4512-01 | IS2 |
| TCGA-86-8278-01 | IS2 |
| TCGA-78-7148-01 | IS4 |
| TCGA-55-8302-01 | IS4 |
| TCGA-62-A46P-01 | IS3 |
| TCGA-55-8091-01 | IS2 |
| TCGA-MP-A4TD-01 | IS2 |
| TCGA-86-A4JF-01 | IS1 |
| TCGA-55-8616-01 | IS3 |
| TCGA-78-7540-01 | IS3 |
| TCGA-95-A4VP-01 | IS2 |
| TCGA-55-6970-01 | IS4 |
| TCGA-44-3396-01 | IS4 |
| TCGA-50-5072-01 | IS4 |
| TCGA-MP-A4T8-01 | IS1 |
| TCGA-55-6987-01 | IS4 |
| TCGA-95-A4VN-01 | IS4 |
| TCGA-MP-A4T4-01 | IS4 |
| TCGA-78-7146-01 | IS4 |
| TCGA-50-5045-01 | IS4 |
| TCGA-86-A456-01 | IS3 |
| TCGA-49-6745-01 | IS4 |
| TCGA-86-8668-01 | IS3 |
| TCGA-49-AAR9-01 | IS1 |
| TCGA-73-4659-01 | IS4 |
| TCGA-86-8585-01 | IS1 |
| TCGA-78-7537-01 | IS3 |
| TCGA-69-7974-01 | IS4 |
| TCGA-86-8672-01 | IS4 |
| TCGA-49-AAR0-01 | IS3 |
| TCGA-91-6828-01 | IS2 |
| TCGA-44-A4SU-01 | IS3 |
| TCGA-55-6971-01 | IS2 |
| TCGA-62-A46V-01 | IS3 |
| TCGA-67-4679-01 | IS3 |
| TCGA-05-4424-01 | IS4 |
| TCGA-55-6543-01 | IS2 |
| TCGA-49-AARE-01 | IS1 |
| TCGA-86-7701-01 | IS4 |
| TCGA-49-AARQ-01 | IS1 |
| TCGA-44-A479-01 | IS4 |
| TCGA-73-4666-01 | IS4 |
| TCGA-78-7163-01 | IS3 |
| TCGA-55-8621-01 | IS2 |
